# PAREameters: a tool for computational inference of plant miRNA–mRNA targeting rules using small RNA and degradome sequencing data

**DOI:** 10.1093/nar/gkz1234

**Published:** 2020-01-16

**Authors:** Joshua Thody, Vincent Moulton, Irina Mohorianu

**Affiliations:** 1 School of Computing Sciences, University of East Anglia, Norwich NR4 7TJ, UK; 2 Wellcome-MRC Cambridge Stem Cell Institute, University of Cambridge, Cambridge CB2 0XY, UK

## Abstract

MicroRNAs (miRNAs) are short, non-coding RNAs that modulate the translation-rate of messenger RNAs (mRNAs) by directing the RNA-induced silencing complex to sequence-specific targets. In plants, this typically results in cleavage and subsequent degradation of the mRNA. Degradome sequencing is a high-throughput technique developed to capture cleaved mRNA fragments and thus can be used to support miRNA target prediction. The current criteria used for miRNA target prediction were inferred on a limited number of experimentally validated *A. thaliana* interactions and were adapted to fit these specific interactions; thus, these fixed criteria may not be optimal across all datasets (organisms, tissues or treatments). We present a new tool, PAREameters, for inferring targeting criteria from small RNA and degradome sequencing datasets. We evaluate its performance using a more extensive set of experimentally validated interactions in multiple *A. thaliana* datasets. We also perform comprehensive analyses to highlight and quantify the differences between subsets of miRNA–mRNA interactions in model and non-model organisms. Our results show increased sensitivity in *A. thaliana* when using the PAREameters inferred criteria and that using data-driven criteria enables the identification of additional interactions that further our understanding of the RNA silencing pathway in both model and non-model organisms.

## INTRODUCTION

Improvements to Next Generation Sequencing technologies have resulted in larger and more diverse experiments, including ones that make use of multiple data types, for example, to increase prediction accuracy of regulatory interactions by combining small RNA (sRNA) sequencing and messenger RNA (mRNA) quantification (1). These improvements have also led to the sequencing and annotation of different organisms’ genomes and facilitated functional studies outside of the context of model organisms (2). However, a vast proportion of our understanding of specific biological mechanisms is based on the study of model organisms, mostly due to their lower regulatory complexity and availability of extensive, varied, public sequencing datasets. Many computational methods designed for extracting information and features from sequencing data (e.g. sRNA classification and target prediction) often summarize the data-mining results into rule-based models, derived from experimental observations. However, this approach carries the risk of overfitting a model (e.g. set of thresholds or accepted ranges) on specific sets of observations.

Small RNAs are short, non-coding RNAs with important roles in transcriptional and post-transcriptional gene regulation in eukaryotes (3). In plants, the latter mode of action is achieved via a class of sRNAs, the microRNAs (miRNAs), which reduce the amount of mRNA available for translation by directing the RNA-induced silencing complex (RISC) to their sequence-specific mRNA target(s) and inducing cleavage and subsequent degradation of the mRNA (4). The miRNA classification criteria were first proposed by Ambros *et al.* (5) and Meyers *et al.* (6); however, more recently these criteria have been updated based on a substantial increase in publicly available sequencing datasets and known miRNA annotations by Axtell *et al.* (7). For example, the new miRNA annotation criteria (7) decreased the stringency in the number of allowed mismatches and asymmetric bulges compared to the previous annotation model (5, 6). In this study, we investigate the applicability and portability of the current miRNA target interaction model.

Most miRNA target prediction tools use fixed rule-based targeting criteria, majority of which are variations of the rules inferred by Allen *et al.* (8) on experiment specific, low-throughput validated *A. thaliana* miRNA–mRNA interactions. We briefly overview these tools, starting with approaches relying solely on sequence properties, specifically sequence complementarity within the target duplex, and continuing with tools that incorporate degradome datasets into their model. The former category comprises three webservers: (i) miRU (9), for which the predictions rely on a rule-based model that limits the number of mismatches, G:U pairs and asymmetric bulges; to reduce the false positive rate, the miRU predictions are optionally subject to a conservation analysis to filter those with predicted target sites existing in other genomes; (ii) psRNATarget (10), which uses two sets of criteria for prediction, V1 (10) and V2 (11), the former uses the same scoring system as miRU complemented with an analysis of the target site accessibility using the RNAup (12) program; the latter is based on the V1 criteria with and increased size of the seed region, from 2–8 nt to 2–13 nt based on a previous study (13); (iii) Tapir (14), which uses the FASTA local alignment algorithm (15) to predict duplexes, which are then subject to hybridization analysis using RNAhybrid (16). Criteria similar to those postulated in Allen *et al.* (8) are implemented in TargetFinder (17) in which the rule-based scoring system is used in conjunction with a Smith–Waterman alignment algorithm in the FASTA package (15) to find valid miRNA–mRNA duplexes. One particularly prominent problem with fixed, sequence-based targeting criteria are how they address miRNA–mRNA target sites that contain central mismatches (7), e.g. psRNATarget classifies all interactions containing central mismatches as translational repression ones (10, 11). However, this contradicts the more refined set of potential outcomes illustrated in the literature, namely that central mismatches can induce mRNA cleavage (8), act as target-mimics (18, 19), cause translational repression (20) or simply be non-functional (21). Thus, without additional data it is difficult to predict miRNA function based solely on complementarity patterns.

One such type of additional data is Parallel Analysis of RNA ends (PARE) sequencing (22), also known as degradome sequencing, which captures the 5′ ends of downstream cleaved mRNAs and can be used to quantitatively predict miRNA–mRNA interactions (23). Tools for predicting miRNA targets that combine the Allen *et al.* criteria, with minor variations, and degradome sequencing data, in chronological order, are CleaveLand4 (23), PAREsnip (24), sPARTA (25) and PAREsnip2 (26). The performance of these tools on the model plant *A. thaliana* was recently compared in a previous study (26), using an updated set of low-throughput, experimentally validated interactions obtained by combining interactions previously published in the literature (24, 27, 28) and entries from miRTarBase (29) with any duplicates removed. The performance evaluation, over three biological replicates, demonstrated that even the most sensitive tool, PAREsnip2, was only able to capture ∼80% of the expressed and experimentally validated interactions when using the Allen *et al.* criteria. Further analyses revealed that the remaining ∼20% were missed mostly due to discrepancies in the number or position of mismatches, gaps, G:U pairs and the minimum free energy (MFE) ratio.

These results suggest that the current target criteria may be too stringent or over-fitted on a small set of organism, tissue or treatment specific experimentally validated miRNA–mRNA interactions. Analyses of miRNA–mRNA interactions in various organisms have shown that currently implemented criteria do not capture all known and expressed miRNA–mRNA interactions (e.g. in *A. thaliana* (30) and *O. sativa* (31)). This is further borne out by our analysis, where we show that, by following a similar approach for manually inferring targeting criteria as Allen *et al.* (Supplementary Table S1), we achieve a sensitivity increase of ∼15% when evaluating on a more extensive set of experimentally validated interactions in *A. thaliana* (Supplementary Table S2). In addition, the portability of current criteria across organisms and tissues has not yet been quantitatively evaluated. Furthermore, the sensitivity and precision of a set of predictions may differ based on the size or characteristics of the input data. For example, the functional analysis of a specific miRNA may benefit from reduced precision, yet good sensitivity, to increase the number of candidates for further investigations; whereas an analysis on the entire set of sRNAs requires concerted high sensitivity and precision.

In this study, we propose a new tool, PAREameters, for data-driven inference of plant miRNA targeting criteria. Using publicly available sequencing datasets, we illustrate how PAREameters extracts information from paired sRNA and degradome sequencing data, in conjunction with miRNA annotations (e.g. from miRBase (32)), to infer criteria that results in an increase in sensitivity when evaluated in *A. thaliana*. We show that different subsets of miRNA–mRNA interactions, such as those containing conserved or species-specific miRNAs, those found in monocots and dicots, and those identified in model and non-model organism, display variation in their target interaction properties. The tool is freely available, open source and provided as part of the UEA sRNA Workbench (33).

## MATERIALS AND METHODS

### The PAREameters pipeline

**Figure 1.**
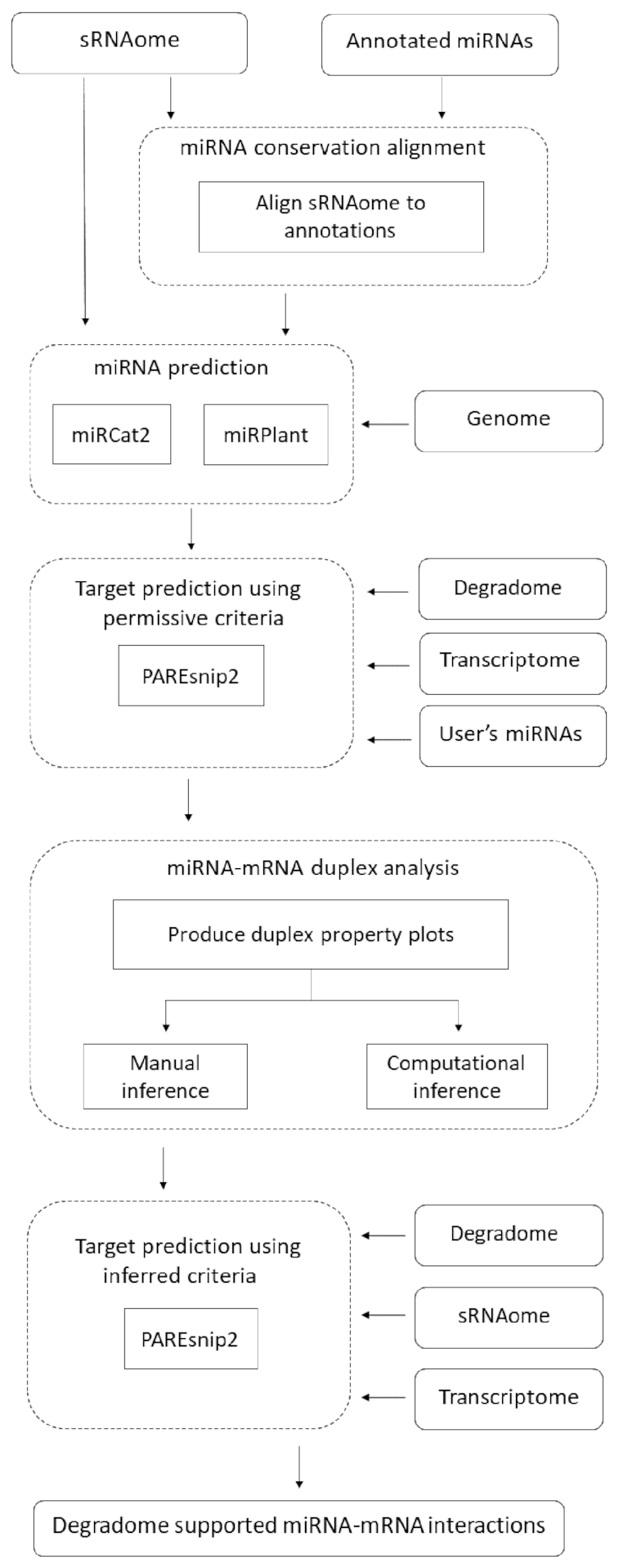
PAREameters pipeline. The input and output data are represented by continuous rounded rectangles, processes are represented by straight rectangles and the different steps of the analysis are represented by dashed rounded rectangles. PAREameters takes as input two types of sequencing samples, paired sRNA and degradome, a genome with corresponding annotations and current miRBase miRNA annotations. The output is a set of data-inferred thresholds for a rule-based prediction of miRNA–mRNA interactions using e.g. PAREsnip2. The sRNAome and degradome inputs are the experiment-specific datasets whereas the genome, transcriptome and annotated miRNA inputs are part of the species annotation.

In Figure 1, we present an overview of the PAREameters pipeline. The input consists of synonymous sRNA and PARE samples; technical or biological replicates can be used for assessing technical variation and noise between samples or for the exclusion of spurious results. An annotated reference genome and transcriptome, and a set of known plant miRNAs (e.g. from miRBase (32)) are also required. The tool’s output consists of miRNA predictions and their mRNA targets, based on a set of highly permissive parameters (Supplementary Table S1). PAREameters also provides a set of suggested targeting criteria, based on these predictions, but also provides the properties of these interactions as individual outputs. In doing so, the user can interpret the results manually to infer criteria that satisfy their sensitivity and precision requirements.

The first stage of the pipeline is to remove low quality reads, sequencing errors or to identify sample outliers, PAREameters includes several optional filtering methods: (i) sequences containing ambiguous bases (e.g. Ns) are discarded; (ii) a low sequence complexity filter is applied based on the single, di- or tri-nucleotide frequencies (26), with set thresholds of 75%, 37.5% and 25%, respectively; (iii) all reads that do not align to the provided reference genome are discarded. We now explain each of the other stages of the pipeline in more detail.

### miRNA prediction

The miRNA candidates used as input for PAREameters are obtained via two approaches: (i) with focus on conserved miRNAs, the input sRNA samples are aligned (+/+ strand only) to all known plant miRNA sequences, obtained from miRBase (32), allowing up to two mismatches and no gaps. The selected sequences are then used as input to miRPlant (34). Candidates that fulfill the criteria for miRNA prediction (default parameters) are then retained for the subsequent steps; (ii) with focus on all miRNAs (conserved and new) as predicted using miRCat2 (35) (default plant parameters) with the whole sRNA sample as input. All data pre-processing required to run the miRNA prediction tools, such as building the bowtie index and organizing the sequencing data into non-redundant format, are handled by PAREameters.

### Target prediction using permissive criteria

Small RNAs that satisfy miRNA biogenesis criteria (as described above) are provided as input to PAREsnip2 (26). Also to compensate the stringent criteria of miRNA prediction tools, the user can provide their own annotated miRNA entries if they have an abundance ≥5 (user-defined parameter) but did not fulfill the criteria of the prediction tools. The target prediction is then performed on the input data using a set of highly-permissive, user-configurable, parameters (Supplementary Table S1).

The miRNA–mRNA interactions predicted by PAREsnip2 are kept if the abundance of the peak of interest is ≥5 and are further classified into high-confidence (HC) or low-confidence (LC). For the former, the peak is the highest across the whole transcript (i.e. category 0 or 1); for the latter, the peak is not the highest on the transcript (i.e. category 2 or 3). The categorization of miRNA–mRNA interactions used for this study is based on the distribution of abundances of the degradome reads aligned to each transcript, as previously described (23–26). Briefly, peaks of categories 0 to 3 correspond to reads with abundance >1: category 0 peaks correspond to reads with maximum abundance on a transcript where there is only one maximum; similarly, category 1 peaks indicate the read with the maximum abundance on a transcript, for which there is more than one maximum; category 2 and category 3 peaks correspond to reads with abundance above and below the average read abundance on the transcript, respectively. Peaks with abundance <5 are excluded, because it is difficult to distinguish between true miRNA cleavage products and random degradation at such low abundance.

When comparing the results of PAREameters, where similar results were observed for all replicates, only one was randomly selected to illustrate the conclusions for all the subsequent comparative analyses.

### miRNA–mRNA duplex analysis and inference of targeting criteria

Valid miRNA–mRNA duplexes, based on the analysis of the degradome data coupled with the miRNA prediction, are characterized using specific properties, such as the number and location of mismatches, G:U wobble pairs and adjacent mismatches, the alignment score and the minimum free energy (MFE) ratio. The algorithm then infers a set of targeting criteria that attempts to retain at least 85% (user-defined parameter) of the valid miRNA–mRNA duplexes. We chose the default value of the retain rate parameter based on the analysis of sensitivity gain against precision loss of inferred criteria across an incremental range of retain rate values on a benchmark leaf *A. thaliana* dataset comprising three replicates (26), presented in the results. The biological interpretation of the retain rate threshold is that a higher degree of complementarity between a miRNA and its target results in higher confidence that the interaction is genuine, whereas interactions with weaker complementarity may require further experimental validation.

Using a set of experimentally validated interactions as validation (26), we focused on HC interaction pairs at known target sites with corresponding miRNAs (32). The validation classes, true positives (TP), false positives (FP) and positives (P) are used in a loose sense, i.e. TP consists of the predicted interactions with experimental validation, FP is the set of predicted interaction for which, currently, there is no experimental validation, and P is the set of experimentally validated interactions with corresponding HC peaks. For each set of targeting rules, we present the sensitivity as Se = TP / P (proportion of predicted validated interactions) and the precision as PPV = TP / (TP+FP) (proportion of validated interactions, out of the total number of reported interactions). In our evaluation, we did not include specificity as a measure of performance because the class of true negatives (TN) cannot be accurately determined. The set of TN comprises the interactions for which there is experimental evidence that interactions do not occur; since the current available information is based on positive events, i.e. experimental validation confirming the interaction happens within an experimental context, it is not possible to obtain a comprehensive set of TN data. Moreover, degradome based miRNA target prediction tools are validation-driven, i.e. they only report interactions that are predicted to be TP based on the defined criteria, which makes it impossible to perform the specificity calculation as perceived TN results are not reported.

In addition, PAREameters provides a summary of the interaction properties, enabling the manual curation of the results and allowing the user to choose a set of targeting criteria that satisfies their choice of sensitivity and precision.

The significance of the distribution of properties with respect to a reference set of miRNA–mRNA interactions was calculated using offset χ^2^ tests and the contribution of each feature was assessed using individual Fisher exact tests (36) (e.g. when comparing conserved versus species-specific interactions, the former is considered the reference). The χ^2^ tests were used to assess the overall differences in distributions, across all 21 positions, whereas the Fisher exact tests compared the values for each individual position, against the sum of values for all remaining 20 positions. Finally, the relative distributions of miRNA–mRNA duplex MFE ratios (8, 26) were analyzed using Kolmogorov–Smirnov tests; briefly, the distributions were first sampled, without replacement, to the same number of entries (given the high number of measurement present in each of compared subsets, this did not distort the original MFE distributions); next, the cumulative distributions were directly compared using the Kolmogorov–Smirnov test and the *P*-value was reported. The significance threshold for all statistical tests was set at 0.05

### Implementation of PAREameters

The PAREameters tool was implemented in Java (version 8); the code used to create the plots and perform the significance tests is implemented in R (version 3.5.1, Apple Darwin) and is invoked from the PAREameters pipeline using system calls, assuming a valid version of R is installed and correctly configured. All computational analyses and benchmarking were performed on a desktop machine running Ubuntu 18.04 equipped with a 3.40GHz Intel Core i7–6800K six core CPU and 128GB RAM. PAREameters is optimized both in runtime and computational resource usage; the analysis of a typical *A. thaliana* and *T. aestivum* sample completes in ∼30 min and 1 day 10 h, with 6 and 10 GB memory (RAM) requirements, respectively. PAREameters is a user-friendly, cross-platform (Windows, Linux and MacOS) application that enables users to analyze sequencing datasets without the need of specialized support or dedicated hardware.

### Datasets

Three *A. thaliana* datasets were used comprising paired sRNA and PARE samples: (D1) wild-type leaf triplicates (D1A, D1B and D1C), GSE90771 (sRNAs) (35) and GSE113958 (PARE) (26); (D2) wild-type leaves in a growth time-series at 35 days (D2A), 45 days (D2B) and 50 days (D2C), GSE55151 (37); (D3) wild-type flower (D3A), leaf (D3B), root (D3C) and seedling (D3D) of plants grown at 15°C, NCBI BioProject PRJNA407271 (38). The genome and transcriptome versions are TAIR10 and were obtained from The Arabidopsis Information Resource (39). The set of experimentally validated *A. thaliana* miRNA–mRNA interactions were obtained from a previous study (26).

In addition to the *A. thaliana* datasets, we exemplify the usage of PAREameters on sRNA and corresponding PARE datasets from *A. trichopoda* leaf (D4A) and opened female flower (D4B) (GSE41811), *G. max* leaf (D5) (GSE76636) (40), *O. sativa* inflorescence (D6) (GSE18251) (41) and *T. aestivum* 2.2mm spikes (D7) (GSE36867) (42). The transcriptome and genome sequences for organisms other than *A. thaliana* were obtained from EnsemblPlants Release 43 (43), namely, *A. trichopoda* genome version AMTR1.0, annotation version AMTR1.0, *G. max* genome version 2.1, annotation version 2.1, *O. sativa* genome version IRGSP-1.0, annotation version IRGSP-1.0, *T. aestivum* genome version IWGSC (genome build accession GCA_900519105.1), annotation version IWGSC.

Summaries of each dataset, such as the number of raw and unique reads and genome matching reads, are presented in Supplementary Table S4. In addition, for the sRNA data, we report the number of known miRNAs present (based on current miRBase (Release 22) (32) annotation) and for the PARE data, we also include the number of transcriptome matching reads.

## RESULTS

### Evaluation of inferred targeting rules in *A. thaliana*

We first illustrate the differences in sensitivity and precision between two sets of manually inferred criteria in *A. thaliana*. These criteria are those previously defined by Allen *et al.* and those we manually inferred on a more comprehensive set of experimentally validated interactions (26). We then highlight the advantages of the data-driven approach implemented in PAREameters by presenting the increase in sensitivity of the computationally inferred targeting rules compared with the Allen *et al.* criteria when benchmarked on multiple *A. thaliana* datasets.

Using the *A. thaliana* leaf dataset (D1), we employed two sets of targeting criteria, the Allen *et al.* criteria and criteria we manually inferred from a set of validated *A. thaliana* miRNA–mRNA interactions (26) (Supplementary Table S1). These criteria were provided as input parameters for PAREsnip2 (26), which is highly configurable and designed to handle the prediction of all sRNA targets from sequencing data (paired sRNA and degradome). The evaluation of these criteria showed an increase in sensitivity from 78.5–81.4% to 94.5–96.2%, with precision values of 88.7–92.1% and 82.1–85.9% for the Allen *et al.* criteria and the manually inferred criteria, respectively (Supplementary Table S2), over three biological replicates. Upon further inspection, the majority of validated interactions that were missed using the manually inferred criteria were due to having an MFE ratio less than the selected cut-off value of 0.65. The MFE ratio quantifies the hybridization strength between the miRNA and its target and thus a higher cut-off value may result in interactions more likely to cause cleavage being reported.

The increase in performance of the manually inferred criteria may be due to over-fitting on the larger set of interactions. In addition, due to the scarcity of validated interactions (either as number of valid interactions or localization of specific modes of action in different cell types (44)), these criteria may not be portable between various organisms or tissues. Therefore, we used the PAREameters tool to infer targeting criteria from the *A. thaliana* D1, D2 and D3 datasets. The resulting criteria (Supplementary Table S5) were then utilized by PAREsnip2 for target prediction and the results evaluated and compared to the predictions obtained using the Allen *et al.* criteria. The evaluation method used is identical to that of the manually inferred criteria. Specifically, for each dataset, the class of positive (P) data included experimentally validated miRNA–mRNA interactions with HC transcript peaks and corresponding miRNA sequence with abundance ≥5.

The results, presented in Table 1, show that the computationally inferred criteria provide increased sensitivity compared to the Allen *et al.* criteria, whilst also maintaining precision on most datasets. Over all datasets, PAREameters inferred criteria with a median sensitivity of 88.5% (range: 82.8–89.4%) versus 81.4% (range: 75.6–84.6%) for the Allen *et al.* criteria. The median precision for the PAREameters inferred criteria was 91.3% (range: 80.1–96.8%) versus 91.4% (range: 83.8–97.5%) for the Allen *et al.* criteria. We also evaluated the time and memory performance of PAREameters on each dataset. The runtime of the pipeline depends on the size of the input data (sequencing depth of the sRNA and PARE samples and the size of the reference genome). On *A. thaliana* D1, D2 and D3 datasets, the runtime range was 16 min and 52 s to 1 h 4 min (this excludes the time taken to build the bowtie index as this is only done once per species) and the memory usage varied between 5GB and 8GB (Supplementary Table S6). The inference component of PAREameters is linear on the size of the sRNA and PARE input data.

**Table 1.** Comparison of sensitivity and specificity between the Allen *et al.* criteria and the PAREameters inferred criteria on the *A. thaliana* datasets

Dataset	# miRNAs	# V	Allen V	Inferred V	Allen NV	Inferred NV	Allen Se	Inferred Se	Allen PPV	Inferred PPV	Se gain	PPV difference
D1A	37	129	105	112	9	8	81.4%	86.8%	92.1%	93.3%	5.4%	1.2%
D1B	38	131	109	116	11	10	83.2%	88.5%	90.8%	92.0%	5.3%	1.2%
D1C	35	121	95	107	12	14	78.5%	88.4%	88.7%	88.4%	9.9%	-0.3%
D2A	40	140	117	125	14	29	83.5%	89.3%	89.3%	81.1%	5.8%	-8.2%
D2B	38	137	113	121	13	30	82.4%	88.3%	89.6%	80.1%	5.9%	-9.5%
D2C	40	144	117	120	3	4	81.2%	83.3%	97.5%	96.7%	2.1%	-0.8%
D3A	32	79	64	68	4	7	81.0%	86.1%	94.1%	90.6%	5.1%	-3.5%
D3B	29	70	57	58	11	13	81.4%	82.8%	83.8%	81.6%	1.4%	-2.2%
D3C	36	111	84	98	6	7	75.6%	88.2%	93.3%	93.3%	12.6%	0.0%
D3D	35	104	88	93	3	3	84.6%	89.4%	96.7%	96.8%	4.8%	0.1%

The PAREameters inferred criteria lead to an increased sensitivity; the apparent loss in precision may be due to the incomplete characterization (and validations) of regulatory interactions and can only increase as more experimentally confirmed interactions become available. V = validated interactions, NV = non-validated interactions, Se = sensitivity and PPV = precision.

#### Evaluation of data input size and retain rate on sensitivity and precision

We now demonstrate that the increase in sensitivity of the PAREameters inferred criteria when compared to the Allen *et al.* criteria is not a result of overfitting on the input data by evaluating performance using a cross-validation approach. We then show how increasing the amount of training data may lead to a more accurate representation of inferred targeting criteria. Finally, we assess how the retain rate parameter impacts sensitivity and precision of the PAREameters inferred criteria.

Based on the properties of HC miRNA–mRNA duplexes with cleavage signal confirmation in the PARE data, PAREameters inferred targeting criteria that increased the sensitivity and retained precision versus existing fixed criteria when tested against the set of experimentally validated interactions in *A. thaliana*. To avoid the overfitting of targeting criteria based on characteristics of the input data, we tested the stability of the inferred properties using a cross-validation technique and the set of experimentally validated *A. thaliana* miRNA–mRNA interactions on the D1, D2 and D3 datasets. Specifically, we used the HC interactions with corresponding miRNA sequences in each dataset as a starting point. We then randomly split the HC validated interactions in each dataset to form two groups: the training group, containing 75% of the data, and the testing group, which contained the remaining 25%. PAREameters was used to infer parameters on the training set and these were employed by PAREsnip2 for target prediction on the test set. We then calculated the sensitivity and precision of the inferred parameters on the training set and on the test set. The random cross-validation was repeated 50× and the results (Supplementary Table S7) show that PAREameters is able to infer targeting parameters with a median sensitivity of 77.5% (range: 67.0–81.3%) and precision 83.2% (range: 75.0–100.0%) when evaluated on the unobserved testing data.

The decrease in sensitivity from our previous analysis likely originates from the fact we are inferring criteria from one set of miRNA–mRNA interactions and testing on a different set of miRNA–mRNA interactions. Whereas previously, we were inferring criteria from the whole set of PAREameters predicted HC miRNA–mRNA interactions. This further supports our hypothesis that miRNAs may have different modes of action or target complementarity requirements and demonstrates that using just one set of fixed criteria may not be sufficient when performing miRNA target prediction.

To investigate further how increasing the amount of training data may lead to a more accurate representation of inferred targeting criteria, we evaluated the computationally inferred criteria produced by PAREameters on different sized subsets of the experimentally validated interactions contained within the D1 datasets. Starting with 10% of the validated data, followed by increments of 10% until the final value of 90%, we used PAREameters to infer criteria on the training subset and then evaluated those criteria on the remaining unseen data. Analysis on each subset was performed 50 times and the results shown in Supplementary Table S8. On each dataset, increasing the amount of training data resulted in an overall increase in sensitivity. Intriguingly, the increase in training data resulted in a decrease in precision. However, this should not be seen as a negative result, as we’ve previously stated, the class FP is the set of predicted interaction for which, currently, there is no experimental validation. Indeed, the current class of positive data is almost certainly incomplete, therefore further experimental validation can only increase the sensitivity and precision values for the inferred criteria.

To assess how changes to the PAREameters retain rate parameter impact sensitivity and precision, we evaluated the computational inferred targeting criteria produced by PAREameters on the D1 dataset with increasing retain rate values. The results of this analysis are shown in Supplementary Table S9 and Supplementary Figure S1. Starting with an initial value of 0.5 and with increments of 0.05 thereafter, we recorded the number of validated and non-validated interactions being captured and determined the differences between Se and PPV for each incremental range. Next, we calculated the absolute value of the ratio between the increases in Se with respect to loss in PPV. For example, the Se and PPV values obtained using a retain rate value of 0.75 on the D1A dataset was 75.2% and 95.1%, respectively, and the Se and PPV values obtained using a retain rate value of 0.80 were 83.7% and 93.9%, respectively. This resulted in a Se increase of 8.5% and a loss in PPV of −1.2% for the 0.75–0.80 range and a Se/PPV ratio of 7.1, specifically, there was a 7.1× increase in Se with respect to the loss in PPV for this range increment. The optimal value for the retain rate parameter is obtained at the first increment range that results in a Se/PPV ratio <1 (i.e. the loss in precision is greater than the increase in sensitivity) (Supplementary Table S3). In the *A. thaliana* D1 data used to exemplify the selection of the retain rate parameter, the first increment range with a Se/PPV ratio <1 was the 0.85–0.90 range, which resulted in the value of 0.85 being selected as the default for the retain rate parameter.

Using the initial value on the D1A dataset, we capture a total of 30 miRNA–mRNA interactions, all of which are experimentally validated interactions. At the other end of the scale, using a retain rate of 1.0 captured 156 interactions, which comprised 128 validated and 28 non-validated. The default parameter value (0.85) captures a total of 120 interactions and provides a sensitivity value of 86.8% and precision value of 93.3%. A visual representation of these results of all three replicates in D1, which show similar results, can be found in Supplementary Figure S1. The increment range of 0.85–0.90 was the first to have a Se/PPV ratio <1 and was consistent across biological replicates. In experiments for which the values vary between samples, we recommend the usage of a consistent threshold across all samples of the experiment.

#### Consistency of attribute distributions and inferred criteria across miRNA subsets in A. thaliana

**Figure 2.**
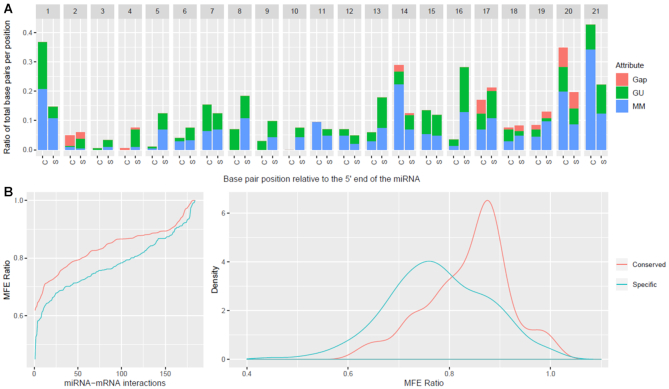
Side-by-side comparison of property distributions for conserved and species-specific miRNAs in *A thaliana*. Using experimentally validated miRNA–mRNA interactions as input, we calculated the position-specific properties (**A**) and the MFE ratio distribution (**B**) for the conserved and species-specific miRNA–mRNA interactions; the former are presented as proportions out of all interactions for each category and the latter as a cumulative distribution. The significance of the differences in the localization of gaps, G:U pairs and mismatches were assessed using offset χ^2^ tests and the contribution of individual categories was evaluated using Fisher exact tests. The first position and the 8–10 range, important for the cleavage ability of the miRNA, showed significant/marginal significant differences; in addition, positions 14 and 16 illustrated the divergence in properties between these subsets. The similarities in the distributions of the MFE ratios were evaluated using the Kolmogorov–Smirnov test, which reported a *P*-value of 8.57 × 10^−10^, the distributions of MFE ratios were different both in location of the mode and the shape of the distributions.

To evaluate the portability of targeting criteria (and distribution of properties) across miRNA subsets, we inferred criteria on a set of conserved and species-specific *A. thaliana* miRNAs (32) and their experimentally validated targets (26). The group built on the conserved miRNAs comprised 201 miRNA–mRNA interactions from 42 unique miRNA sequences (Supplementary Table S10). The group built on miRNAs specific to the *Brassicaceae* family comprised 184 interactions from 47 unique miRNA sequences (Supplementary Table S11). The summaries of the position-specific property distributions, which include the localizations of gaps, mismatch, and G:U wobbles and the MFE ratio distributions for the conserved and specific miRNA interactions are presented in Figure 2 panel A and panel B, respectively. In Figure 2A, the *Brassicaceae* specific miRNAs show highly similar results to that of Allen *et al.* (8), e.g. a large proportion of mismatches or G:U wobble pairs at position 1, no mismatches at the canonical positions 9 and 10 and relatively few mismatches in the 5′ core region (positions 2–13) of the miRNA when compared to the 3′ end. In contrast, the requirements for complementary of species-specific miRNAs (Figure 2A) appear to differ when compared to conserved miRNAs, especially at the miRNA 5′ end, with mismatches being tolerated at positions 5, 8 and 9, in addition to the canonical position 10 of the miRNA.

To evaluate whether the differences in properties between specific-specific and conserved miRNA interactions in *A. thaliana* are significant, we performed χ^2^ tests of significance using the conserved properties as the expected distribution and the species-specific properties as the observed distribution. Additionally, we use the Fisher’s exact test to determine the specific property at each position responsible for the significance of the differences. The results of the significance analysis for the position-specific property distributions are presented in Table 2. Based on the χ^2^ tests, significant differences between properties can be found at positions 1, 5, 8, 14, 16 and 21. Based on the Fisher’s exact test, position 16 has significant differences in both proportions of mismatches and G:U pairs, positions 5, 8, 14, 20 and 21 have significant differences in proportion of mismatches and positions 1 and 13 have significant differences in the proportion of G:U pairs. We also analyzed the differences in MFE ratio distributions between conserved and species-specific miRNAs, shown in Figure 2B, and the significance of the differences were evaluated using a Kolmogorov–Smirnov test, which reported a *P*-value of 8.57 × 10^−10^. These results may suggest a higher complementarity requirement between conserved miRNAs and their targets than that of species-specific miRNAs.

**Table 2.** χ^2^ and Fisher’s exact test significance results on the position-specific properties for conserved and species-specific miRNA–mRNA interactions in *A. thaliana*. The contribution of specific properties, such as mismatch (MM), G:U pair and gap is assessed using Fisher exact tests. The first position and the 8–10 nt region (which is important for inducing cleavage) are either significant or marginally significant, indicating a potential divergence in the type of interactions of either conserved or species-specific miRNAs

	χ^2^	MM	G:U	Gap
1	**0.00**	0.08	**0.01**	1.00
2	0.58	1.00	0.36	0.72
3	0.71	1.00	0.62	1.00
4	0.13	1.00	0.06	1.00
5	**0.02**	**0.03**	0.11	0.99
6	0.71	0.99	0.27	0.99
7	0.77	1.00	0.43	1.00
8	**0.01**	**0.00**	1.00	1.00
9	0.34	0.11	0.53	0.99
10	0.18	0.11	0.21	0.99
11	0.56	0.43	0.62	1.00
12	0.76	0.49	0.99	1.00
13	0.10	0.25	0.10	1.00
14	**0.02**	**0.00**	1.00	0.68
15	0.99	1.00	0.99	1.00
16	**0.00**	**0.00**	**0.00**	1.00
17	0.36	0.48	0.43	0.27
18	0.59	0.74	0.49	0.62
19	0.42	0.19	0.68	1.00
20	0.10	**0.04**	0.59	1.00
21	**0.00**	**0.00**	0.81	1.00

To investigate the portability between criteria inferred exclusively on conserved or species-specific miRNA interactions, we evaluated the inferred rules of each set of interactions (all four pairwise combinations: conserved rules on conserved interactions, conserved rules on species-specific interactions and the similar pairs on the species-specific rules), using PAREsnip2. The results, presented in Table 3, show a consistent decrease in sensitivity for both the conserved and species-specific miRNAs when inferring criteria on the other subset of miRNA–mRNA interactions. Specifically, a decrease from 82.1% to 65.7% and 76.1% to 56.0% for the conserved and species-specific miRNA–mRNA interactions, respectively. Further investigation into these differences support our previous observation regarding the differences in MFE ratio of conserved and species-specific miRNA interactions, with the inferred values being 0.75 and 0.68, respectively, further supporting our previous observation regarding an increased complementarity requirement for conserved miRNAs. Another intriguing difference between the inferred criteria is an allowed mismatch or G:U pair at position 10 of the species-specific miRNAs.

**Table 3.** Sensitivity on cross pairwise comparisons for criteria inferred on conserved or species-specific miRNAs for the validated *A. thaliana* interactions. The targeting criteria were inferred using a retain rate of 0.85; a considerable decrease in sensitivity was observed for the mismatched pairs i.e. training on conserved and testing on specific and the symmetric pair, highlighting the impact of data-inferred targeting criteria.Parameter

Inferred interactions	Evaluated interactions	Possible	Captured	Sensitivity
Conserved	Conserved	201	165	82.1%
Specific	Conserved	201	132	65.7%
Specific	Specific	184	140	76.1%
Conserved	Specific	184	103	56.0%

The differences between the properties of conserved and species-specific miRNA–mRNA interactions highlight the need for customization in the set of criteria used for describing and capturing miRNA–mRNA interactions when conserved or species-specific miRNAs are involved.

### Evaluation of miRNA targeting criteria in non-model organisms

Current miRNA targeting rules, inferred on interactions, mostly consisting of conserved miRNAs from *A. thaliana* (8), have been applied to other species for target prediction (45–48). However, to the best of our knowledge, no comprehensive investigation into the suitability of these fixed targeting criteria has been performed in non-model organisms. The characterization of miRNA–mRNA interactions has been facilitated by both the increased complexity of experiments involving non-model plant species and through the analysis of RNA degradation profiles (PARE (22) sequencing and more recently NanoPARE (49)), which despite technical limitations (e.g. sequencing bias (50)) can provide reliable high-throughput pseudo-validation of microRNA-mediated cleavage sites.

**Figure 3.**
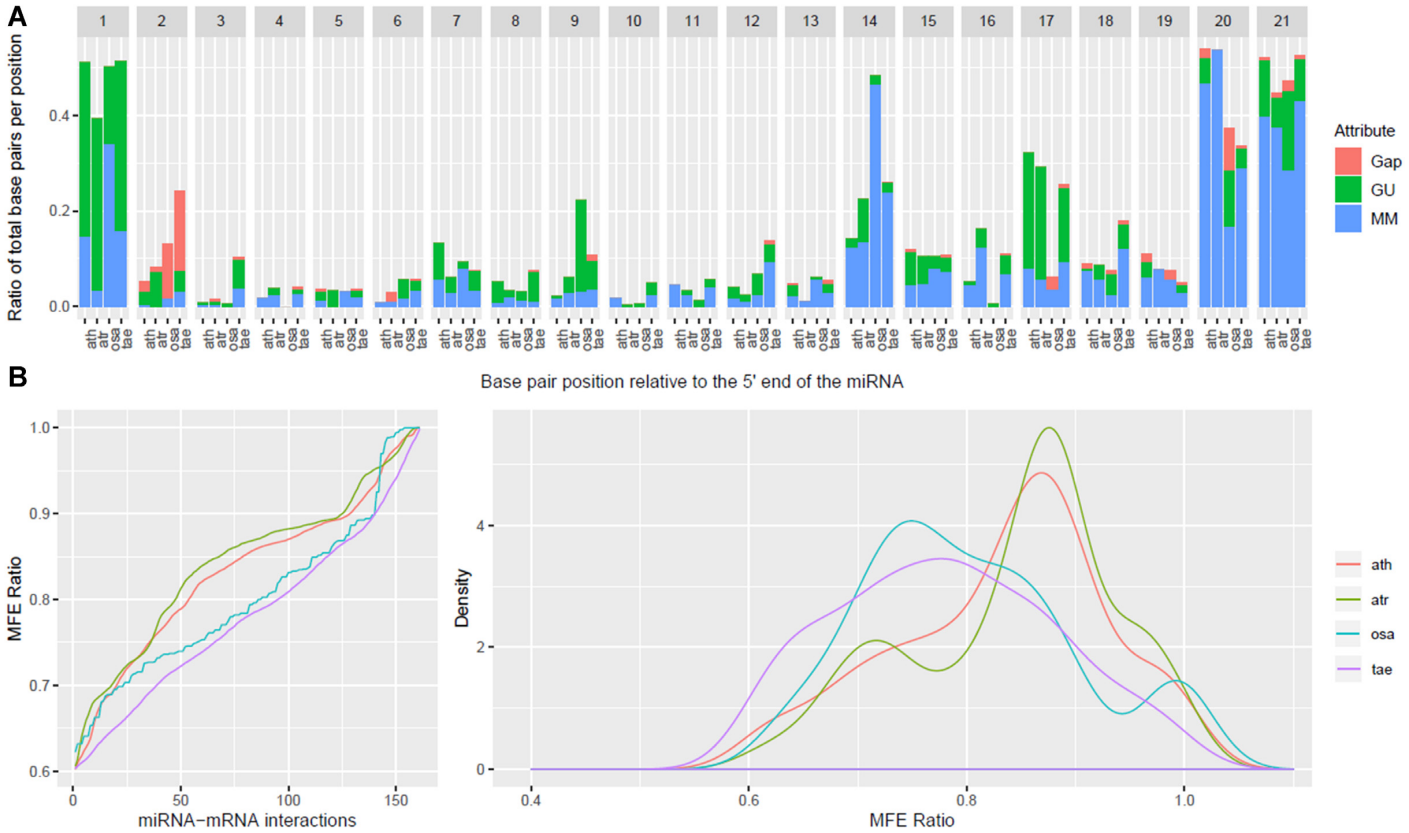
Side-by-side comparison of flower miRNA–mRNA interaction property distributions in monocots and dicots. The position-specific properties (**A**) and MFE ratio distribution (**B**) of miRNA–mRNA interactions from flower tissues in *A. thaliana*, *A. amborella*, *O. sativa* and *T. aestivum*. The position-specific properties showed significant differences at certain positions and there is a clear separation in the MFE distributions between monocots and dicots. The differences in properties for particular organisms from the current *A. thaliana* inferred criteria (e.g. MMs at position 1 for *A. amborella*, G:U pairs at position 9 for *O. sativa*, and almost perfect complementarity at positions 16–17 for *O. sativa*) and the distinction observed for the MFE ratios support the hypothesis that species or tissue specific, and data-inferred criteria may reveal a more accurate set of miRNA–mRNA regulatory interactions.

To investigate the suitability and portability of the fixed *Allen et al*. criteria on non-model organisms and evaluate the scope for customized, organism-specific rules, we conducted an exploratory analysis using as input the HC degradome-supported miRNA–mRNA interactions reported by PAREameters. We compared the inferred rules for flower and leaf tissues in several organisms to produce a quantitative summary of the variation ranges of thresholds for the selected rules. Table 4 shows these summaries of inferred criteria per organism; Figure 3A illustrates the position-specific distributions of G:U pairs, mismatches and gaps, and Figure 3B shows the MFE ratio distributions for the miRNA–mRNA duplexes from flower tissue across organisms in *A. thaliana*, *A. trichopoda*, *O. sativa* and *T. aestivum*. Similar plots for leaf tissue in *A. thaliana*, *A. trichopoda* and *G. max* are presented in Supplementary Figure S2.

**Table 4.** Overview of data-inferred thresholds inferred using PAREameters on model and non-model organisms in flower and leaf tissue. Differences in reported thresholds are observed both between organisms (e.g. monocots versus dicots) and between tissues

	Flower tissue				Leaf tissue			
	*A. thaliana* (D3A)	*A. trichopoda* (D4B)	*O. sativa*	*T. aestivum*	*A. thaliana* (D1A)	*A. thaliana* (D2B)	*A. trichopoda* (D4A)	*G. Max*
Allow MM at position 10	Yes	No	No	Yes	Yes	Yes	No	Yes
Allow MM at position 11	Yes	Yes	No	Yes	Yes	Yes	Yes	Yes
Max # adjacent MM in CR	0.00	0.00	0.00	0.00	0.00	0.00	0.00	0.00
Max # MM in CR	1.00	1.00	1.00	1.00	1.00	1.00	1.00	1.00
Max score	4.50	4.50	4.00	5.00	4.50	5.00	4.50	4.50
Max # MM	3.00	3.00	3.00	3.00	3.00	3.00	3.00	3.00
Max # G:U	2.00	2.00	2.00	2.00	2.00	2.00	2.00	2.00
Max # adjacent MM	1.00	1.00	0.00	1.00	1.00	1.00	1.00	1.00
MFE ratio cut-off	0.72	0.72	0.70	0.66	0.69	0.65	0.71	0.69

The distributions of position-specific properties in flower tissue show interesting variations between species. To evaluate whether the non-model organism distributions differ from the *A. thaliana* distributions, we used the offset χ^2^ test and a localized Fisher’s exact test (Table 5). The former show significant differences at position 1, positions 1, 2, 9, 14, 17 and 20, and positions 2, 3 and 20, for *A. trichopoda*, *O. sativa* and *T. aestivum*, respectively. The results of the localized Fisher’s exact test show significant differences between mismatches at position 1, positions 1, 14, and 20, and positions 14 and 20, for *A. trichopoda*, *O. sativa* and *T. aestivum*, respectively, supporting the conclusion that species specific, data driven criteria could facilitate a better description of the miRNA–mRNA interactions. Additionally, the tests show significant differences between G:U pairs at positions 1, 7, 9 and 17 in *O. sativa* and significant differences in gaps at position 2 for both *O. sativa* and *T. aestivum*.

**Table 5.** χ^2^ and Fisher’s exact test significance results on the position-specific properties for non-model organisms versus *A. thaliana* in flower tissue. The first position and the ninth suggest that subtle differences do exist between *A. thaliana* and other organisms in key positions that determine the selection and mode of action for miRNAs

Species	*A. trichopoda*				*O. sativa*				*T*. aestivum			
Position	χ^2^	MM	GU	Gap	χ^2^	MM	GU	Gap	χ^2^	MM	GU	Gap
1	**0.03**	**0.00**	1.00	1.00	**0.00**	**0.00**	**0.00**	1.00	0.99	1.00	1.00	1.00
2	0.66	1.00	0.37	1.00	**0.03**	0.62	0.36	**0.02**	**0.00**	0.36	1.00	**0.00**
3	1.00	1.00	1.00	1.00	0.95	0.99	1.00	0.99	**0.05**	0.21	0.06	0.99
4	0.95	1.00	0.62	0.99	0.79	0.62	1.00	1.00	0.91	0.99	1.00	1.00
5	0.92	0.62	1.00	0.99	0.64	0.68	0.36	0.99	0.94	1.00	1.00	1.00
6	0.79	1.00	1.00	0.62	0.39	1.00	0.21	1.00	0.61	0.44	0.36	0.99
7	0.38	0.53	0.25	1.00	0.18	0.61	**0.05**	1.00	0.55	0.53	0.40	1.00
8	0.68	1.00	0.44	1.00	0.91	1.00	0.72	1.00	0.94	1.00	0.56	0.99
9	0.56	0.99	0.36	1.00	**0.00**	0.99	**0.00**	1.00	0.12	0.72	0.06	1.00
10	0.79	0.62	1.00	1.00	0.71	0.62	1.00	1.00	0.79	1.00	0.36	0.99
11	0.84	0.72	1.00	1.00	0.38	0.21	1.00	1.00	0.79	1.00	0.62	1.00
12	0.93	1.00	1.00	1.00	0.9	1.00	0.49	0.99	0.15	0.08	0.72	1.00
13	0.73	0.68	0.36	0.99	0.61	0.33	0.68	1.00	0.92	1.00	1.00	1.00
14	0.22	0.83	0.04	0.99	**0.00**	**0.00**	1.00	1.00	0.19	**0.04**	1.00	1.00
15	0.98	0.99	0.79	0.99	0.33	0.40	0.21	1.00	0.50	0.56	0.25	1.00
16	0.10	**0.05**	0.44	1.00	0.43	0.21	1.00	1.00	0.53	0.56	0.44	1.00
17	0.95	0.61	1.00	1.00	**0.00**	0.40	**0.00**	0.62	0.51	.001	0.16	0.99
18	0.49	0.79	0.36	1.00	0.12	0.13	0.21	1.00	0.18	0.49	0.11	1.00
19	0.37	0.79	0.36	0.62	0.59	1.00	0.21	1.00	0.53	0.53	0.99	0.62
20	0.16	0.39	0.11	0.62	**0.00**	**0.00**	0.14	0.08	**0.02**	**0.01**	0.99	0.62
21	0.44	0.88	0.23	1.00	0.28	0.10	0.43	0.62	0.83	0.77	0.65	1.00

In addition to the position-specific properties, the MFE ratio was also investigated as a discriminative feature (Figure 3B); the Kolmogorov–Smirnov test was used to evaluate differences between distributions of different species. The distribution of MFE ratios and results of the statistical test, presented in Supplementary Table S12, illustrates the differences between monocots and dicots, with significant differences only reported when comparing different groups.

The differences observed between conserved and specific-specific miRNAs in *A. thaliana* prompted a similar investigation in other, non-model organisms. Similarly, as for *A. thaliana* miRNA interactions, we classified the miRNAs that had HC predicted interactions, as reported by PAREameters, into conserved or species-specific for each of the non-model organisms. Specifically, miRNAs present only in an individual clade, based on current miRBase annotations (Release 22) (32), were considered species-specific; otherwise they were classified as conserved. The conservation analysis was done against the current miRNA variants from miRBase, allowing up to two mismatches (on any positions) and no gaps. If a miRNA predicted on a non-model organism dataset did not match any miRNA variant in miRBase or aligned only to a known species-specific miRNA, then it would be classified as a species-specific, otherwise the miRNA was classified as conserved. The summaries of the position-specific properties distributions and MFE ratio distributions for each of the non-model organisms are presented in Supplementary Figures S3–7. The results of the significance tests comparing the conserved and species-specific properties are presented in Supplementary Tables S13–17.

To illustrate the impact of the differences between targeting properties and subsequently inferred targeting criteria in non-model organisms, we focus on the results in *T. aestivum* (Wheat), presented in Supplementary Figure S7 and Supplementary Table S17. Out of the 21 positions analysed, 9 had significant differences based on the χ^2^ tests (the conserved properties were considered the expected distribution and the species-specific properties were the observed distribution), with three of these differences in the miRNA core region (positions 2, 3 and 12). Also showing a significant difference were the MFE ratio distributions, evaluated using the Kolmogorov–Smirnov test, which reported a *P*-value of 0. Also, other non-model organisms showed significant differences within the miRNA core region, for example, *A. trichopoda* flower (Supplementary Figure S4 and Supplementary Table S14) and *O. sativa* inflorescence (Supplementary Figure S6 and Supplementary Table S16). Moreover, significant differences between the MFE ratio distributions are observed in *A. trichopoda* flower (Supplementary Figure S4 and Supplementary Table S14) and *G. max* leaves (Supplementary Figure S5 and Supplementary Table S15).

#### Employing data-driven targeting criteria on non-model organisms

To evaluate the differences in number and identity of predicted miRNA targets when using the Allen *et al.* and PAREameters inferred criteria on the non-model organisms, we performed target prediction using PAREsnip2. The inferred criteria were able to capture a larger number of interactions; the only exception was observed for the D6 (*O. sativa*) dataset for which 149 interactions from 42 miRNAs were found using the Allen *et al.* criteria and 115 interactions from 33 miRNAs using the inferred rules with an overlap of 100%. The larger number of interactions reported for the D5 (*G. max*) and D7 (*T. aestivum*) datasets when compared to D4 (*A. trichopoda*) and D6 (*O. sativa*) may have arisen from number of repeat regions or duplicated transcripts present within the current genome annotation.

We then investigated the overlap between the miRNAs and their interactions for each set of criteria, presented in Table 6, and concluded that, except for D6 (O*. sativa*), a higher number of miRNAs and their interactions were specific to the inferred criteria, highlighting yet again the distance from the Allen *et al.* criteria. For this analysis, we used the default retain rate of 0.85. To explore its effect on the overlap between the Allen *et al.* criteria and the inferred criteria, we repeated the analysis using a retain rate value of 1, to capture all PAREameters reported HC interactions. All of the captured interactions using the Allen *et al.* criteria were a subset of the interactions captured by the PAREameters inferred criteria when using a retain rate of 1 (Supplementary Table S18); the increase in miRNAs with targets varies between 4 (D6) and 102 (D7) and the increase in reported interactions varies between 12 (D6) and 783 (D7), depending on the organism/dataset in question. These results further suggest that the Allen *et al.* criteria may have been too stringent, or inadequately calibrated for the specific organism or miRNAs in question.

**Table 6.** Intersection analysis of interactions predicted using either the Allen *et al.* rules or the PAREameters inferred rules on various datasets. The number of interactions reported by PAREsnip2 using the Allen *et al.* criteria and the PAREameters inferred criteria on the non-model organisms varies between organisms and tissues; the number in brackets represents the miRNAs and interactions specific to the criteria used and could be used to approximate the accuracy of the prediction on non-model organisms. The exact sensitivity and precision values cannot be computed on non-model organisms due to the lack of a large enough set of validated interactions

Dataset	Allen *et al.* miRNAs	Allen *et al.* interactions	Inferred miRNAs	Inferred interactions
D4A	70 (3)	203 (9)	72 (5)	210 (16)
D4B	66 (2)	174 (4)	68(4)	182 (12)
D5	143 (6)	2118 (64)	143 (6)	2243 (189)
D6	42 (9)	149 (34)	33 (0)	115 (0)
D7	91 (2)	1257 (50)	99 (10)	1417 (210)

## DISCUSSION

In this paper, we describe PAREameters, a novel approach and tool that enables data-driven inference of plant miRNA targeting criteria. Through refining the targeting criteria, the discovery and characterization of new miRNA–mRNA interactions per tissue or organism (both model and non-model) becomes possible. When evaluating the performance of the PAREameters inferred criteria, we observed an increase in sensitivity compared to the Allen *et al.* criteria over all the *A. thaliana* datasets, whilst also maintaining precision on most datasets, when benchmarked against a set of experimentally validated miRNA–mRNA interactions.

The comparison of validated miRNA–mRNA interaction properties between conserved and species-specific miRNAs in *A. thaliana* highlighted interesting and perhaps previously unknown differences. When investigating the features of conserved miRNA interactions, we observed similar patterns to that of Allen *et al.* (8) regarding complementarity in the core region of the miRNA (2–13) and at the canonical position 10. This observation is further supported by a recent study of highly conserved miRNAs in *N. benthamiana* (19), where it was shown that a single mismatch at the 5′ end of miR160 significantly diminished target site efficacy, and two or more consecutive mismatches at the 5′ end fully abolished it. Furthermore, the authors highlighted that a single-nucleotide mismatch at positions 9 and 10, in addition to combinations of mismatches at positions 9, 10 and 11 led to the complete elimination of the responsiveness of miR164. However, the species-specific miRNAs tended to tolerate more flexibility at these positions. These results motivated a similar analysis in non-model organisms and the results of which did mirror the trends observed in *A. thaliana*. However, it is important to emphasize that these result from a series of predictions, and are subject to changes from additional, low throughput validations. Nonetheless, this output highlights, yet again, the potential differences in the range of suitable thresholds used for predicting targets for subsets of miRNAs and reiterate the remark that one set of fixed criteria for inferring miRNA–mRNA targets may not be sufficient.

Throughout this study, we used exclusively the HC interactions, reported by PAREameters, for all comparative analysis. This is in part because the strongest degradation signal on a transcript is likely a result of miRNA cleavage and focusing on this subset of interactions increases the confidence in the prediction results. However, it has been shown that weaker/lower abundance degradation signals may also be caused by miRNAs; these can be captured during target prediction, albeit with lower prediction confidence. These lower abundance signals may be a result of lower miRNA expression, reduced cleavage efficiency or even sequencing bias (50). Indeed, it is also possible that the degradation fragments may not be caused by miRNA cleavage but instead are a result of noise or random degradation of the transcript. It has been shown that real miRNA cleavage sites tend to be conserved across biological replicates and therefore, we further tested the hypothesis that the properties of genuine miRNA–mRNA interactions will be consistent between biological replicates. To investigate this, we re-ran the analysis of the *A. thaliana* dataset D1 dataset, allowing both HC and LC interactions to be reported, and compared the results, across replicates, using the same statistical evaluations, as described in the methods.

The outcome of these analyses, presented in Supplementary Figures S8 and S9, show a consistent decrease in the number of LC interactions reported compared to the number of HC interactions and a higher variability in distributions of properties, across replicates, for the LC interactions. This remark supports our previous observation that genuine miRNA cleavage signals are likely to have the strongest signal (category 0 or 1) on transcripts. The consistency of the MFE ratio distributions and the position-specific properties of HC interactions between replicates is remarkable, with no significant differences in properties reported (Supplementary Table S19), supporting our previous hypothesis that genuine miRNA cleavage sites are conserved between biological replicates. Conversely, when comparing the property distributions of LC interactions between replicates, we observe a higher variation in the proportions of interactions with specific properties, with some positions having significant differences reported by the statistical tests (Supplementary Table S20). We speculate that the cause of these variations is due to the higher proportion of putative false positive predictions, i.e. the category 2 and 3 interactions comprise a combination of genuine target sites and random degradation illustrated by the lower abundance of the transcript degradation signals.

In this study, we also highlighted that targeting criteria inferred on non-model organisms or subsets of interactions are less compatible with current fixed criteria and often lead to a decrease in sensitivity. Given the current, limited understanding of the miRNA–mRNA interactions in various species, it is difficult to propose a biological interpretation of these variations, however, based on the side-by-side analysis of various datasets, we can conclude that a customized selection of parameters may result in a higher precision output that could facilitate a more detailed overview of regulatory interactions and an in-depth assessment of the underlying regulatory networks. Furthermore, the differences observed in the flower tissue between monocots and dicots emphasize the usefulness of data-inferred, species and tissue specific thresholds. We have demonstrated that PAREameters is applicable for a wide variety of experimental designs in both model and non-model organisms and could enable further understanding of the subtle variations in miRNA–mRNA interactions in different species, tissues and treatments. In addition, this novel data-driven approach may enable new discoveries, i.e. regulatory sequences or modes of action, within the RNA silencing pathways.

## DATA AVAILABILITY

PAREameters is available as part of the UEA sRNA Workbench (33); it can be downloaded from http://srna-workbench.cmp.uea.ac.uk/. The source code has been released on GitHub and is accessible at https://github.com/sRNAworkbenchuea/UEA_sRNA_Workbench/.

## Supplementary Material

gkz1234_Supplemental_Files
