# Supplementary material for: PAREameters: a tool for computational inference of plant miRNA–mRNA targeting rules using small RNA and degradome sequencing data
Source: Nucleic Acids Res. 2020 Jan 16;48(5):2258–70. doi: 10.1093/nar/gkz1234 (PMC7049721; doi:10.1093/nar/gkz1234)
Supplement: gkz1234_Supplemental_Files [file gkz1234_supplemental_files.zip › supplementary_figures.pdf]

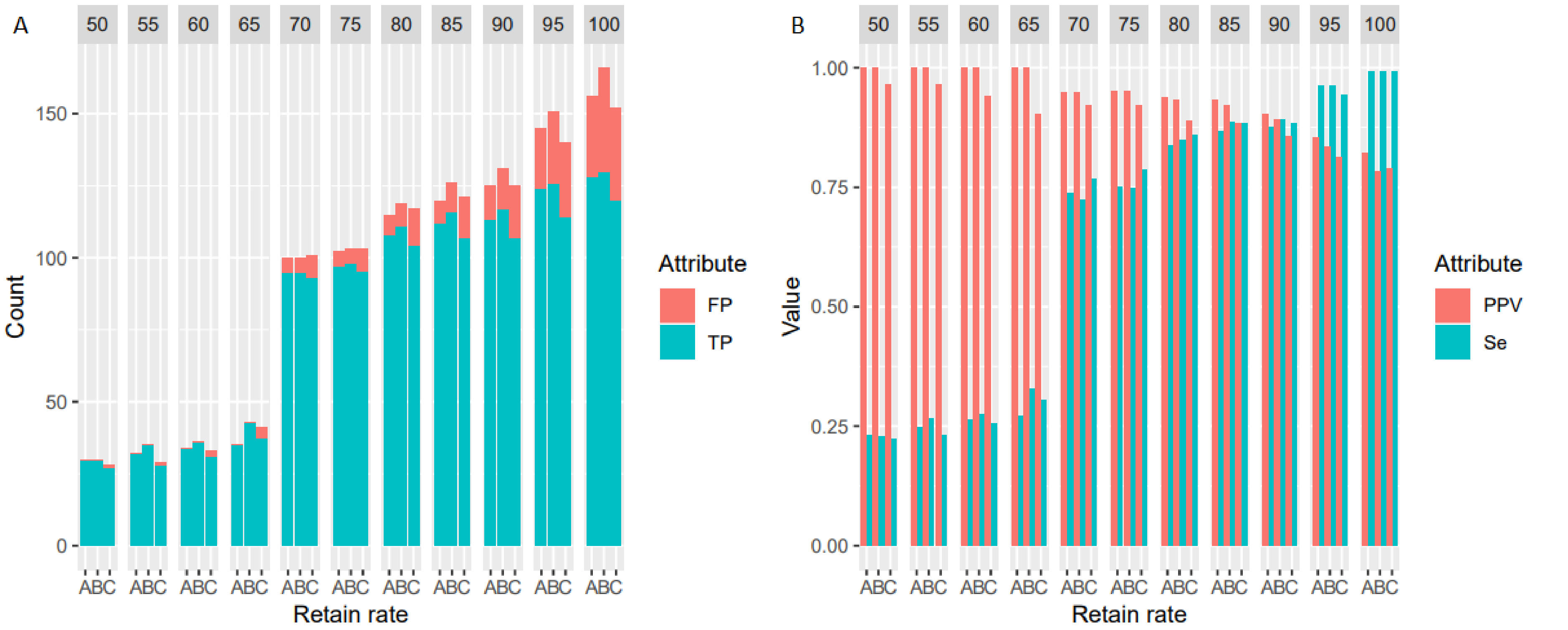

Supplementary Figure S1: **Accuracy (sensitivity and precision) dependence on the retain rate parameter**. Clustered frequency histograms of predicted interactions vs validated/non-validated ones (panel A) and the variation in sensitivity and precision values for increasing values of the retain rate parameter on three A. *Thaliana* leaf replicates, the D1 dataset (panel B) highlight the existence of a data-driven optimum for the retain rate parameter. For this particular dataset the optimum on the Se/PPV ratio is achieved for 0.85. The data-driven optimal value for this parameter is suggested based on the input; however, it still remains a user-configurable parameter. FP = false positive, TP = true positive, A = D1A, B = D1B and C = D1C.

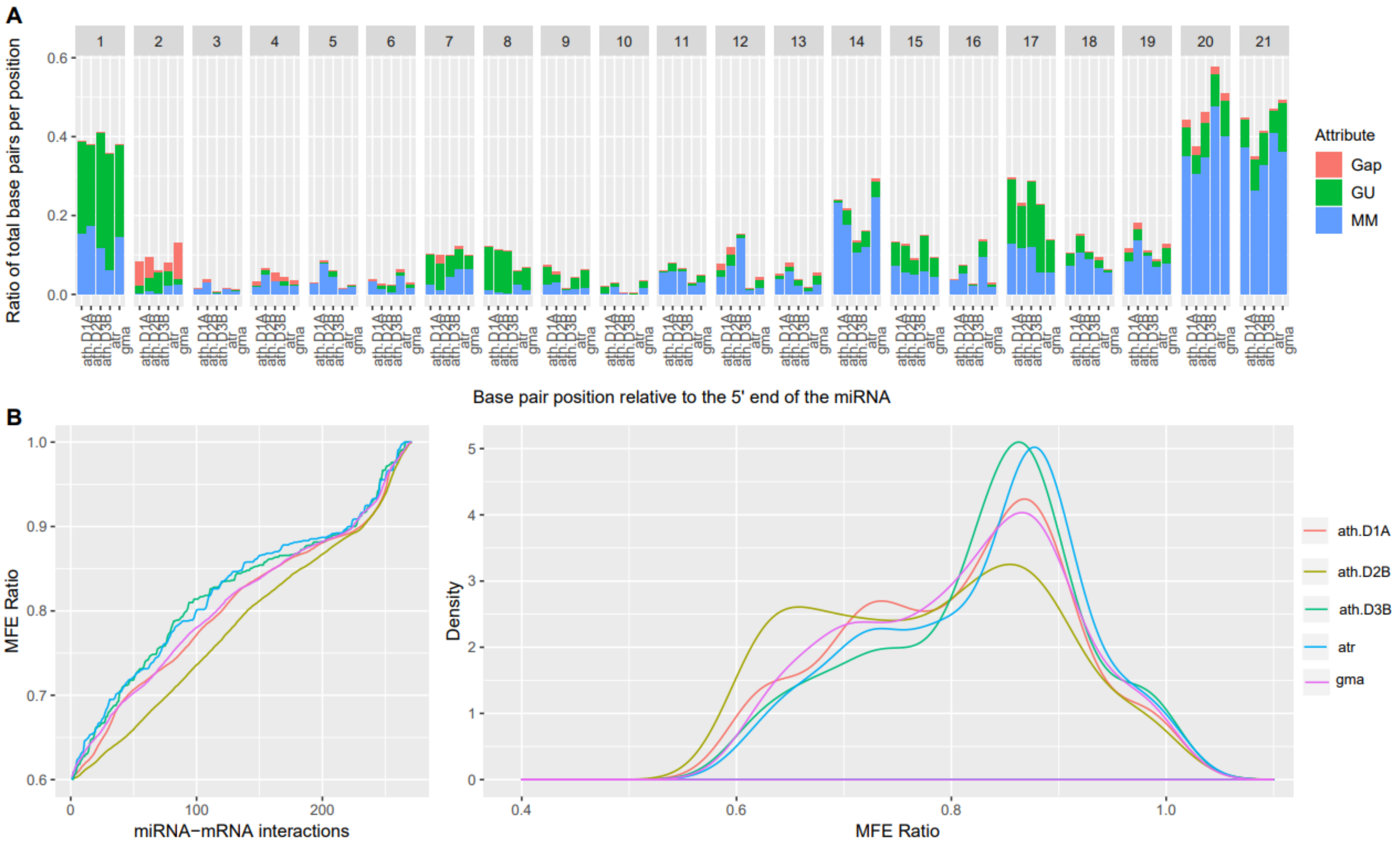

Supplementary Figure S2: **Side-by-side comparison of leaf miRNA-mRNA interaction property distributions in different species and datasets.** The position-specific properties (panel A) and MFE ratio distribution (panel B) of miRNA-mRNA interactions from leaf tissues in *A. thaliana*, *A. amborella* and *G.*

max. The differences in properties for particular organisms and the differences observed for the MFE ratios support the hypothesis that species or tissue specific, and data-driven criteria may reveal a more accurate set of miRNA-mRNA regulatory interactions.

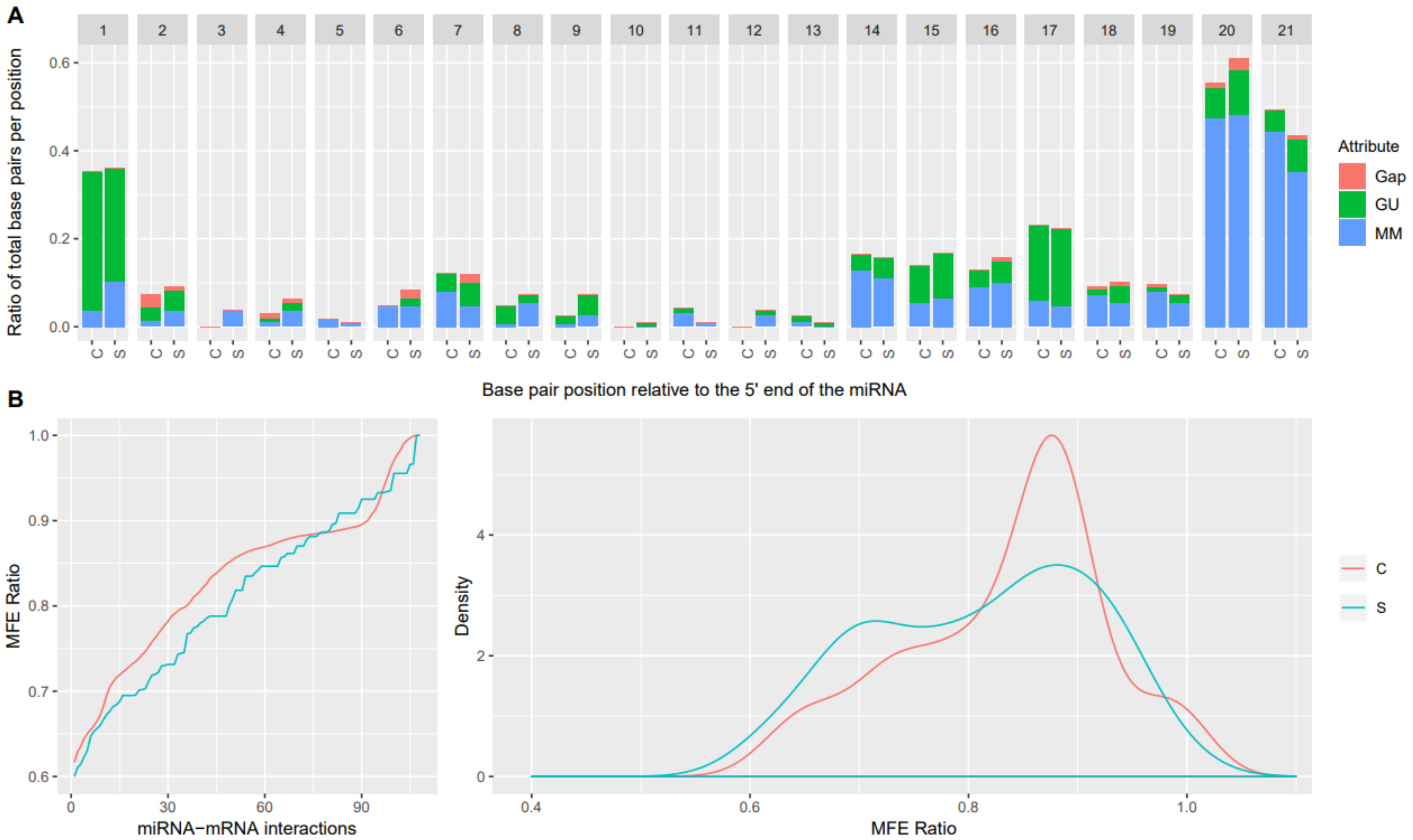

Supplementary Figure S3: **Side-by-side comparison of property distributions for predicted HC interations by conserved and species-specific miRNAs in *A. trichopoda* leaf.** Using PAREamters HC predicted miRNA-mRNA interactions as input, we calculated the position-specific properties (panel A) and the MFE ratio distribution (panel B) for the conserved and species-specific miRNA-mRNA interactions; the former are presented as proportions out of all interactions,

in each category and the latter as a cumulative distribution. The similarities in the distributions of the MFE ratios were evaluated using the Kolmogorov-Smirnov test, which reported a *p*-value of 0.1376412.

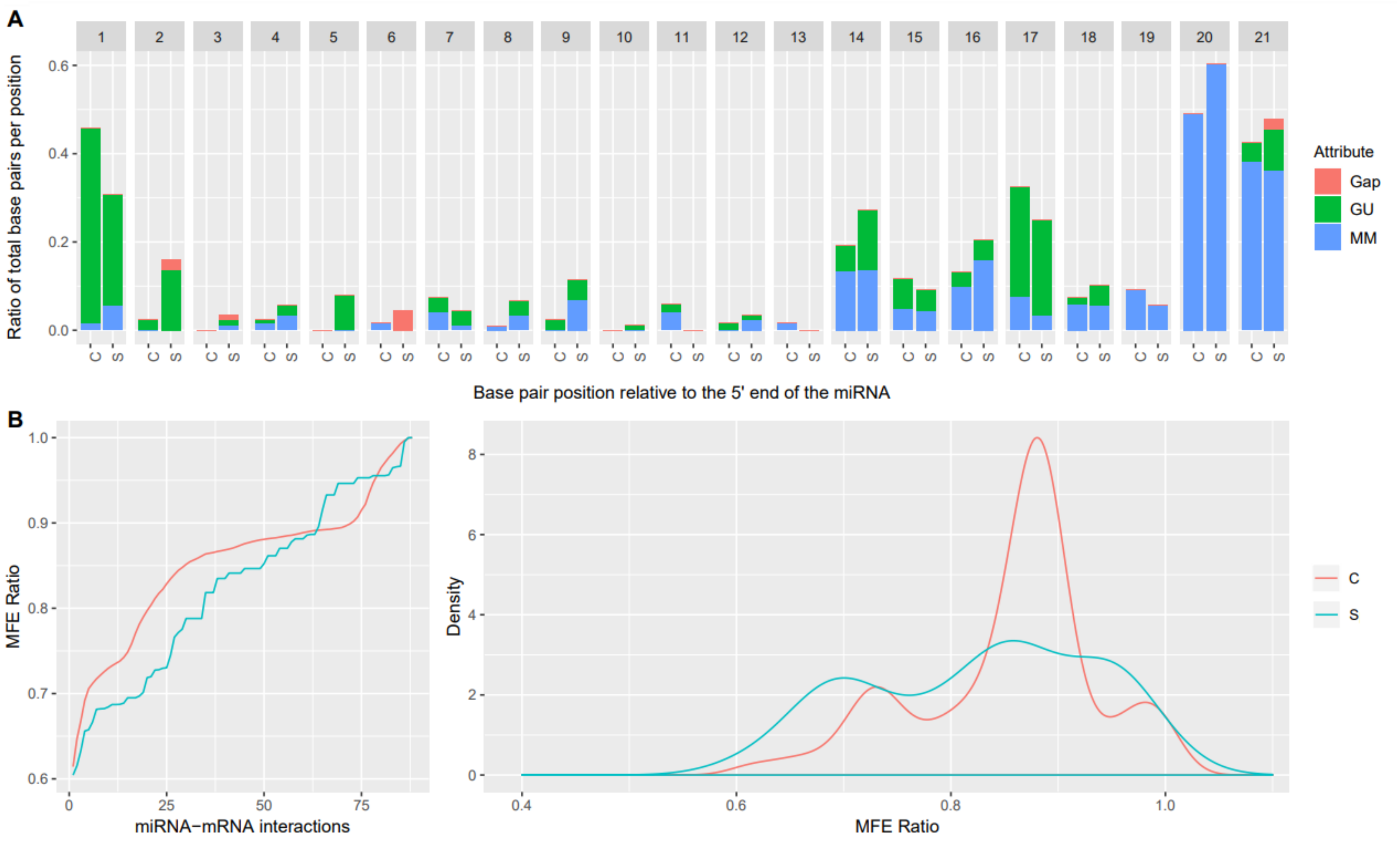

Supplementary Figure S4: **Side-by-side comparison of property distributions for predicted HC interations by conserved and species-specific miRNAs in *A. trichopoda* flower.** Using PAREamters HC predicted miRNA-mRNA interactions as input, we calculated the position-specific properties (panel A) and the MFE ratio distribution (panel B) for the conserved and species-specific miRNA-mRNA interactions; the former are presented as proportions out of all interactions,

in each category and the latter as a cumulative distribution. The similarities in the distributions of the MFE ratios were evaluated using the Kolmogorov-Smirnov test, which reported a *p*-value of 0.01332362.

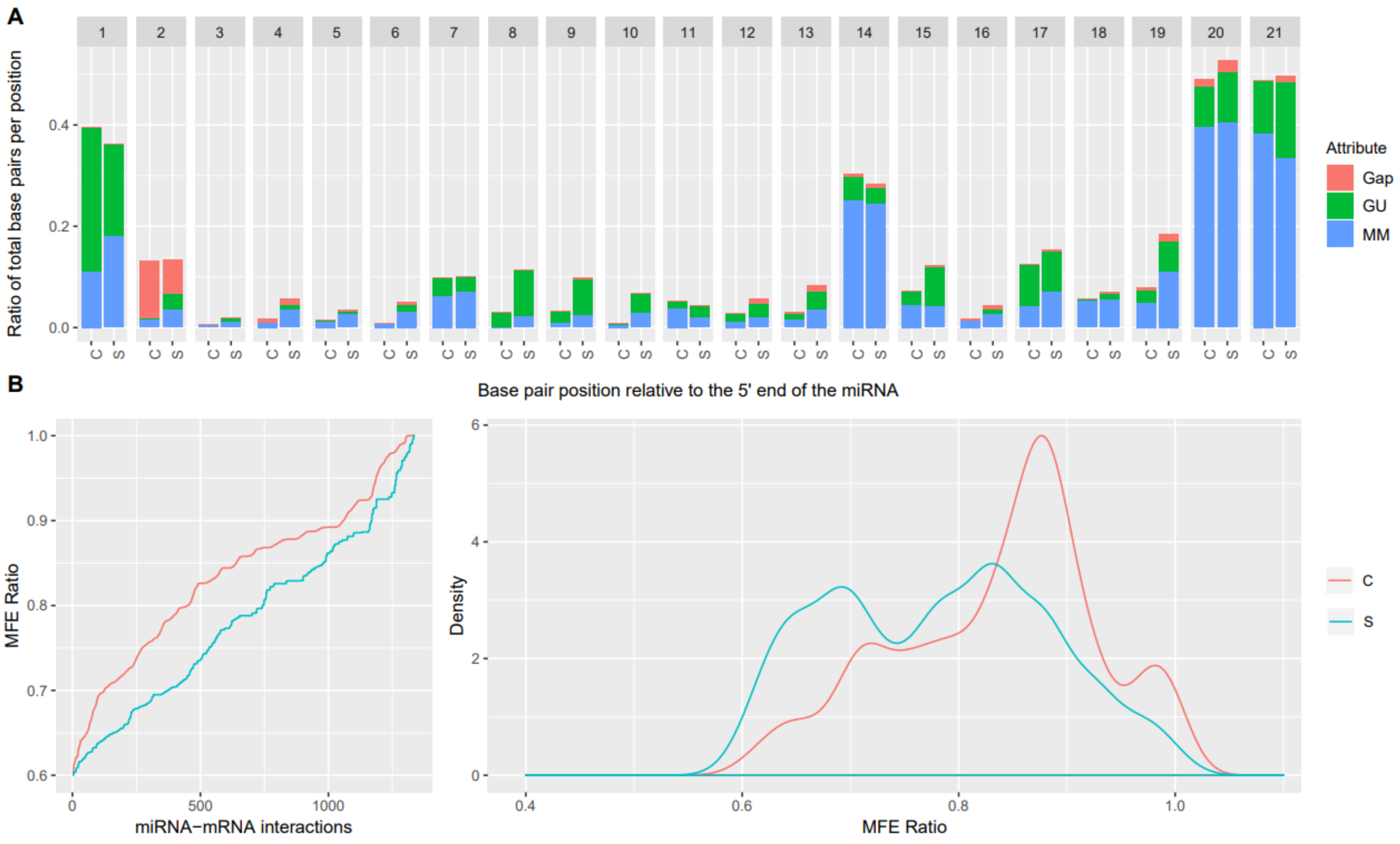

Supplementary Figure S5: **Side-by-side comparison of property distributions for predicted HC interations by conserved and species-specific miRNAs in *G. max* leaf.** Using PAREamters HC predicted miRNA-mRNA interactions as input, we calculated the position-specific properties (panel A) and the MFE ratio distribution (panel B) for the conserved and species-specific miRNA-mRNA interactions*;* the former are presented as proportions out of all interactions, in

each category and the latter as a cumulative distribution. The similarities in the distributions of the MFE ratios were evaluated using the Kolmogorov-Smirnov test, which reported a *p*-value of 0.

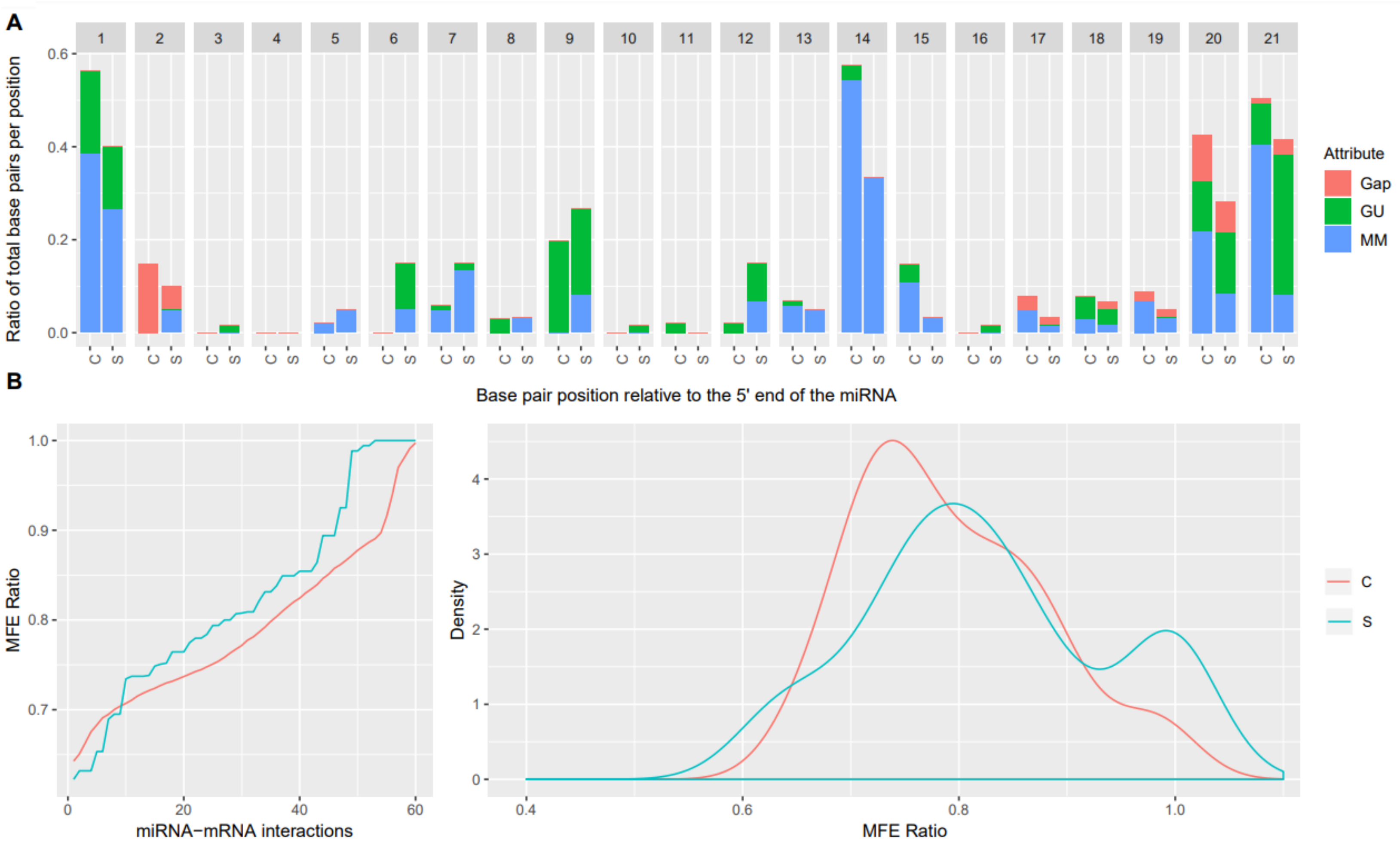

Supplementary Figure S6: **Side-by-side comparison of property distributions for predicted HC interations by conserved and species-specific miRNAs in *O. sativa* inflorescence.** Using PAREamters HC predicted miRNA-mRNA interactions as input, we calculated the position-specific properties (panel A) and the MFE ratio distribution (panel B) for the conserved and species-specific miRNA-mRNA interactions*;* the former are presented as proportions out of all

interactions, in each category and the latter as a cumulative distribution. The similarities in the distributions of the MFE ratios were evaluated using the Kolmogorov-Smirnov test, which reported a *p*-value of 0.2655691.

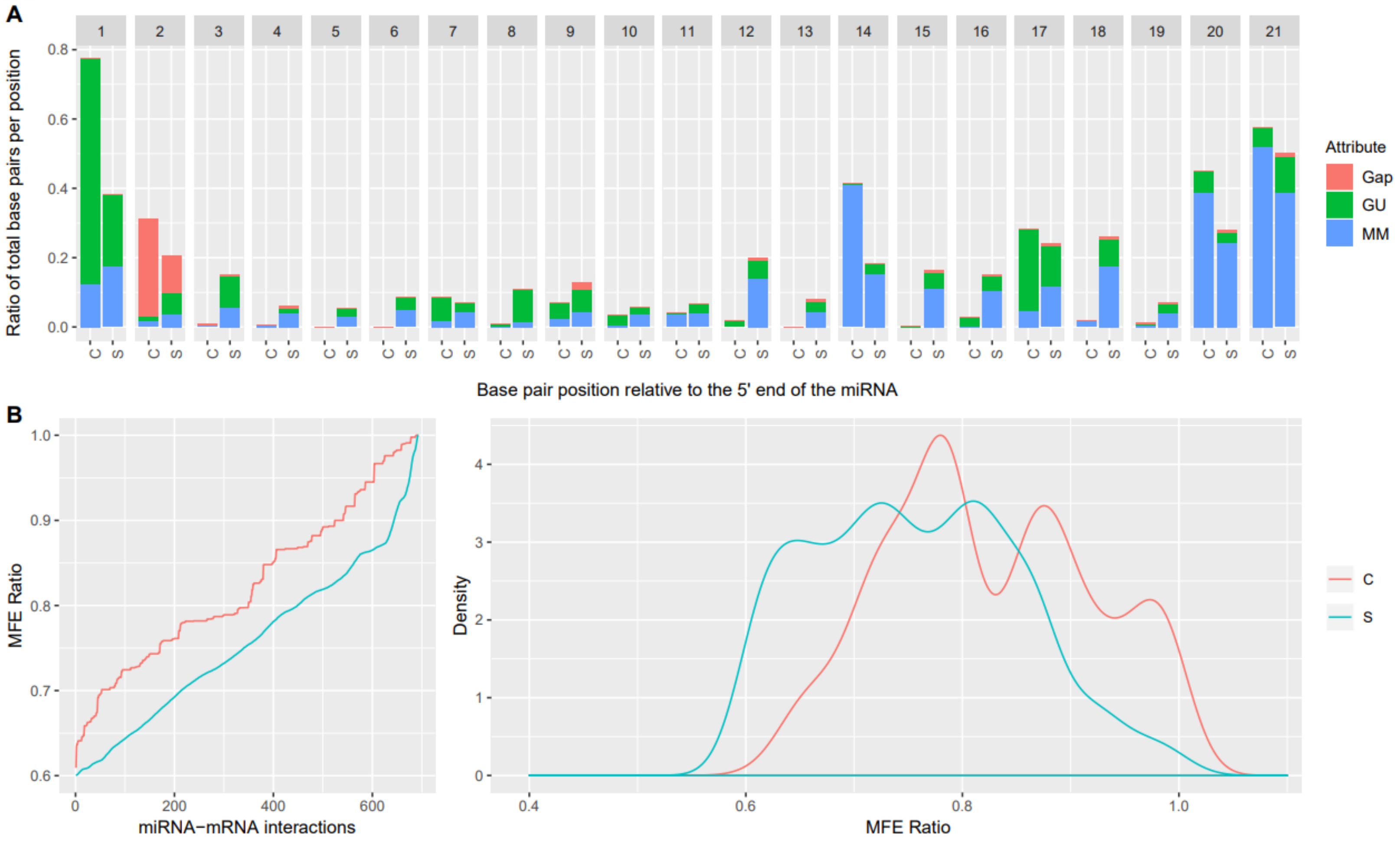

Supplementary Figure S7: **Side-by-side comparison of property distributions for predicted HC interations by conserved and species-specific miRNAs in *T. aestivum* spikes.** Using PAREamters HC predicted miRNA-mRNA interactions as input, we calculated the position-specific properties (panel A) and the MFE ratio distribution (panel B) for the conserved and species-specific miRNA-mRNA interactions; the former are presented as proportions out of all interactions,

in each category and the latter as a cumulative distribution. The similarities in the distributions of the MFE ratios were evaluated using the Kolmogorov-Smirnov test, which reported a *p*-value of 0.

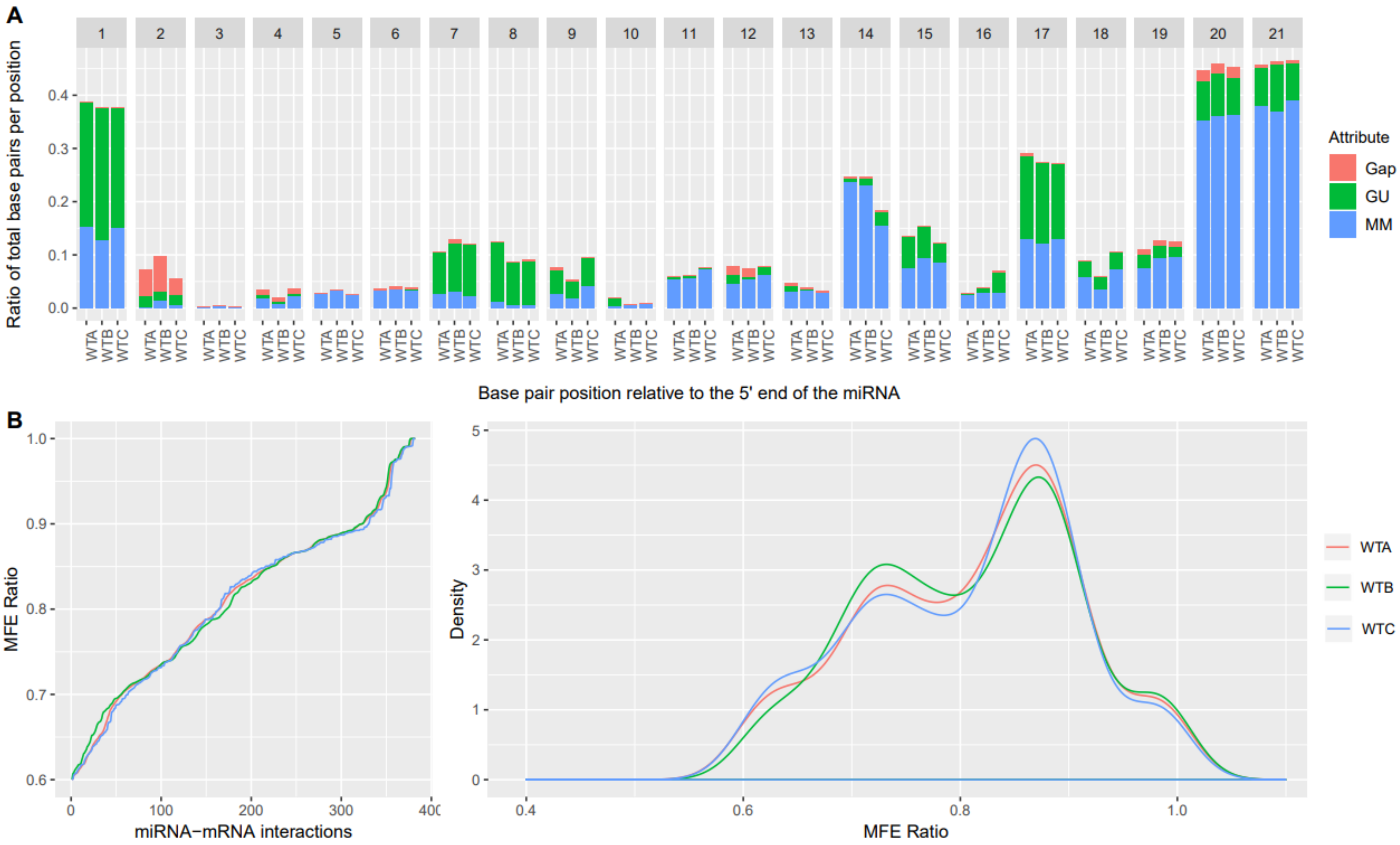

Supplementary Figure S8: **Side-by-side comparison of property distributions for predicted HC interations in D1A (WTA), D1B (WTB) and D1C (WTC).** Using PAREamters HC predicted miRNA-mRNA interactions as input, we calculated the position-specific properties (panel A) and the MFE ratio distribution

(panel B) for the conserved and species-specific miRNA-mRNA interactions*;* the former are presented as proportions out of all interactions, in each category and the latter as a cumulative distribution. The similarities in the distributions of the MFE ratios were evaluated using the Kolmogorov-Smirnov test, which reported a *p*-value of 0.9994413, 0.9973924 and 0.9596333 for D1A vs D1B, D1A vs D1C and D1B vs D1C, respectively.

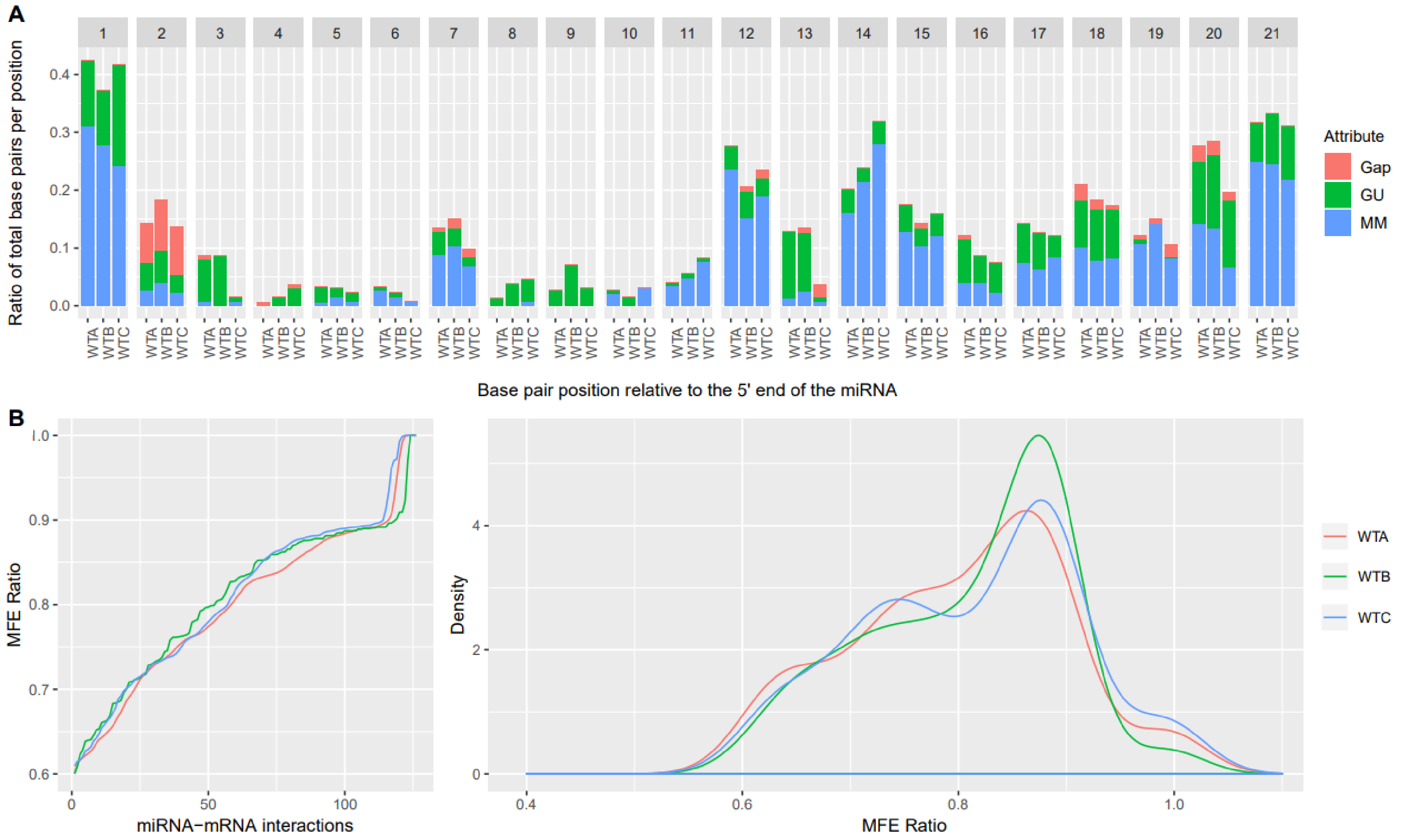

Supplementary Figure S9: **Side-by-side comparison of property distributions for predicted LC interations in D1A (WTA), D1B (WTB) and D1C (WTC).**

Using PAREamters LC predicted miRNA-mRNA interactions as input, we calculated the position-specific properties (panel A) and the MFE ratio distribution (panel B) for the conserved and species-specific miRNA-mRNA interactions*;* the former are presented as proportions out of all interactions, in each category and the latter as a cumulative distribution. The similarities in the distributions of the MFE ratios were evaluated using the Kolmogorov-Smirnov test, which reported a *p*-value of 0.418178, 0.617195 and 0.7229624 for D1A vs D1B, D1A vs D1C and D1B vs D1C, respectively.
