# Supplementary material for: PAREameters: a tool for computational inference of plant miRNA–mRNA targeting rules using small RNA and degradome sequencing data
Source: Nucleic Acids Res. 2020 Jan 16;48(5):2258–70. doi: 10.1093/nar/gkz1234 (PMC7049721; doi:10.1093/nar/gkz1234)
Supplement: gkz1234_Supplemental_Files [file gkz1234_supplemental_files.zip › supplementary_tables.pdf]

Parameter	*Allen et al.*	Manually inferred	Permissive PAREameters criteria
Allow MM at position 10	No	Yes	Yes
Allow MM at position 11	No	Yes	Yes
Max # adjacent MM in CR	1	0	2
Max # MM in CR	2	2	3
Max score	4	5	6
Max # MM	4	4	6
Max # G:U	4	3	5
Max # adjacent MM	2	1	4
MFE ratio cut-off	0.7	0.65	0.6

Supplementary Table S1: **The parameter values for the *Allen et al.*, manually inferred and PAREameters permissive criteria, derived from the experimentally validated *A. thaliana* miRNA-mRNA interactions.** The *Allen et al.* criteria were inferred in 2005 on a small set of experimentally validated interactions, in *A. Thaliana*; the manually inferred criteria were inferred on a larger set of experimentally validated interactions, for the same organism. The permissive parameters are used initially by PAREameters to find HC interactions. The inferred criteria are then extracted from those using the retain rate parameter. MM = mismatch, CR = core regions (positions 2-13 of miRNA), MFE = minimum free energy.

Dataset	# miRNAs	# Validated in dataset	Allen V	Inferred V	Allen NV	Inferred NV	Allen Se	Inferred Se	Allen PPV	Inferred PPV	Se gain	PPV difference
D1A	37	129	105	122	9	20	81.40%	94.57%	92.11%	85.92%	13.18%	-6.19%
D1B	38	131	109	126	11	25	83.21%	96.18%	90.83%	83.44%	12.98%	-7.39%
D1C	35	121	95	115	12	25	78.51%	95.04%	88.79%	82.14%	16.53%	-6.64%
D2A	40	140	117	133	14	43	83.57%	95.00%	89.31%	75.57%	11.43%	-13.75%
D2B	38	137	113	129	13	43	82.48%	94.16%	89.68%	75.00%	11.68%	-14.68%
D2C	40	144	117	138	3	11	81.25%	95.83%	97.50%	92.62%	14.58%	-4.88%
D3A	32	79	64	77	4	10	81.01%	97.47%	94.12%	88.51%	16.46%	-5.61%
D3B	29	70	57	66	11	17	81.43%	94.29%	83.82%	79.520%	12.86%	-4.31%
D3C	36	111	84	106	6	12	75.68%	95.50%	93.33%	89.83%	19.82%	-3.50%
D3D	35	104	88	101	3	7	84.62%	97.12%	96.70%	93.52%	12.50%	-3.19%

Supplementary Table S2: **Sensitivity and precision values for the *Allen et al.* and manually inferred criteria over all the *A. Thaliana* datasets.** An increase in the achieved sensitivity is observed for the manually inferred criteria; the decrease in precision is likely due to the lack of low-throughput validations for a subset of interactions, for which a clear signal is observed in the PARE datasets. Allen = *Allen et al.* criteria, Inferred = the manually inferred criteria on a larger set of validated interactions, V = validated, NV = non-validated, Se = sensitivity and PPV = precision

Transition	D1A			D1B			D1C		
	Sensitivity gain	Precision difference	Ratio	Sensitivity gain	Precision difference	Ratio	Sensitivity gain	Precision difference	Ratio
0.50-0.55	1.5%	0.0%	-	3.8%	0.0%	-	0.8%	0.2%	4.0
0.55-0.60	1.6%	0.0%	-	0.8%	0.0%	-	2.5%	-2.7%	0.9
0.60-0.65	0.7%	0.0%	-	5.3%	0.0%	-	5.0%	-3.7%	1.4
0.65-0.70	46.5%	-5.0%	9.3	39.7%	-5.0%	7.9	46.3%	1.9%	24.4
0.70-0.75	1.6%	0.1%	16.0	2.3%	0.2%	11.5	1.6%	0.1%	16.0
0.75-0.80	8.5%	-1.2%	7.1	9.9%	-1.9%	5.2	7.5%	-3.3%	2.3
0.80-0.85	3.1%	-0.6%	5.2	3.9%	-1.2%	3.3	2.4%	-0.5%	4.8
0.85-0.90	0.8%	-2.9%	0.3	0.7%	-2.8%	0.3	0.0%	-2.8%	0.0
0.90-0.95	8.5%	-4.9%	1.7	6.9%	-5.9%	1.2	5.8%	-4.2%	1.4
0.95-1	3.1%	-3.5%	0.9	3.0%	-5.1%	0.6	5.0%	-2.4%	2.1

Supplementary Table S3: **Sensitivity gain vs precision loss and the absolute ratio between them for values of the retain rate parameter in the 0.50-1.00 range, over three *A. thaliana* leaf replicates (D1 dataset).** The sensitivity and precision were evaluated on the experimentally validated miRNA-mRNA interactions in *A. thaliana* that had corresponding HC transcript peaks within the degradome dataset. The ratio is defined as the absolute value of Se/PPV. The optimal value is obtained for the last transition that results in a Se/PPV ratio ≥ 1 (i.e. there is an equal or greater gain in sensitivity with respect to the loss in precision) and for the D1 dataset, this value was obtained at the 0.80-0.85 transition.

Dataset	Raw reads	Unique reads	Genome matched	% matched	miRNAs present
D1A	6664998	1342846	1006022	74.92%	230
D1B	4492236	1093344	818760	74.89%	213
D1C	5148552	1106222	837919	75.75%	230
D2A	27870710	1935025	1091177	56.40%	252
D2B	26211889	908368	525503	57.90%	239
D2C	28700595	568633	240613	42.31%	209
D3A	18460973	3797561	2534869	66.75%	200
D3B	10408796	1633730	1101861	67.44%	186
D3C	19946757	2837304	1647286	58.06%	211
D3D	6645127	1176424	836656	71.12%	141
D4A	18092450	1765893	656968	37.20%	153
D4B	25781233	4560684	2685317	58.88%	161
D5	33230948	2370300	1641914	69.27%	309
D6	4029462	1991942	1991720	99.99%	227
D7	12541386	5178587	4325043	83.51%	168

Supplementary Table S4A: **Summary statistics of the sRNA sequencing data.** The number of raw (redundant), unique and genome matched reads. In order for an annotated miRNA to be considered present, it must have had an abundance >=5 within the library.

Dataset	Raw reads	Unique reads	Transcriptome matched (+ strand)	% matched
D1A	44871978	11114549	8802080	79.19%
D1B	34315808	10103690	8176679	80.93%
D1C	25588818	7715251	6251721	81.03%
D2A	115224802	19930692	10565361	53.01%
D2B	107999423	19470487	11187427	57.46%
D2C	191294550	7275123	1734835	23.85%
D3A	5263291	2463251	2131493	86.53%
D3B	4809175	2300541	1998764	86.88%
D3C	12666325	3975280	3454112	86.89%
D3D	53840936	9032093	6824080	75.56%
D4A	19990216	4305009	1940976	45.09%
D4B	12609502	3992618	1679932	42.08%
D5	26251057	7704474	6230026	80.86%
D6	4426044	2505523	2268297	90.53%
D7	35477509	14363576	10366761	72.17%

Supplementary Table S4B: **Summary statistics of the degradome sequencing data.** The number of raw (redundant), unique and transcriptome (positive strand only) matching reads within each degradome library.

Parameter	D1A	D1B	D1C	D2A	D2B	D2C	D3A	D3B	D3C	D3D
Allow MM at position 10	Yes	Yes	Yes	Yes	Yes	Yes	Yes	Yes	Yes	Yes
Allow MM at position 11	Yes	Yes	Yes	Yes	Yes	Yes	Yes	Yes	Yes	Yes
Max number of adjacent MM in CR	0	0	0	0	0	0	0	0	0	0
Max number of MM in CR	1	1	1	1	1	1	1	1	1	1
Max score	4.50	4.50	5.00	5.00	5.00	4.50	4.00	4.50	4.50	4.50
Max number of MM	3	3	3	3	3	3	3	3	3	3
Max number of G:U	2	2	2	2	2	2	2	2	2	2
Max number of adjacent MM	1	1	1	1	1	1	1	1	1	1
MFE ratio cut-off	0.69	0.70	0.69	0.66	0.65	0.67	0.72	0.71	0.70	0.71

Supplementary Table S5: **The PAREameters inferred criteria for each of the Arabidopsis datasets.** MM = mismatch, CR = core region (2-13nt of miRNA) and MFE = minimum free energy.

Dataset	Unique sRNAs	Unique PARE seqs	Run Time (hh:mm:ss)	Memory (GB)
D1A	1342846	11114549	00:18:51	6
D1B	1093344	10103690	00:17:41	6
D1C	1106222	7715251	00:17:11	6
D2A	1935025	19930692	00:31:18	8
D2B	908368	19470487	00:38:09	8
D2C	568633	7275123	01:04:24	8
D3A	3797561	2463251	00:18:15	5
D3B	1633730	2300541	00:16:52	5
D3C	2837304	3975280	00:20:30	5
D3D	1176424	9032093	00:17:59	5
D4A	1765893	4305009	01:30:15	6
D4B	4560684	3992618	01:34:12	6
D5	2370300	7704474	02:00:26	7
D6	1991942	2505523	00:41:43	5
D7	5178587	14363576	34:37:10	10

Supplementary Table S6: **The timing and memory usage results for the PAREameters analysis of the Arabidopsis datasets.** The size of the input (as unique entries) and complexity of the underlying genome are the main drivers for both run-time and resource usage.

Dataset	Test Inferred SE	Test Inferred PPV
D1A	81.3%	83.0%
D1B	78.1%	83.3%
D1C	80.0%	82.1%
D2A	79.0%	78.0%
D2B	77.0%	87.3%
D2C	67.0%	89.0%
D3A	68.4%	78.0%
D3B	77.0%	75.0%
D3C	78.0%	96.0%
D3D	76.0%	100%

Supplementary Table S7: **The median sensitivity and PPV (precision) values for the cross-validation experiments on the Arabidopsis datasets.** The CV was done on a 75/25% split for training and testing, respectively. Each analysis was repeated 50 times and the median value was recorded.

Training size (% of total)	D1A Sensitivity	D1A Precision	D1B Sensitivity	D1B Precision	D1C Sensitivity	D1C Precision
0.1	69.0%	95.2%	48.7%	97.2%	71.8%	90.9%
0.2	70.9%	93.8%	59.6%	94.8%	66.7%	90.4%
0.3	77.2%	92.4%	64.3%	94.5%	73.8%	89.4%
0.4	79.2%	91.5%	75.6%	91.9%	70.8%	88.2%
0.5	78.1%	89.9%	76.9%	89.7%	77.5%	86.4%
0.6	75.5%	88.0%	77.9%	87.5%	77.1%	84.1%
0.7	79.0%	85.3%	76.9%	85.3%	77.8%	83.8%
0.8	82.0%	80.8%	80.8%	83.3%	79.2%	82.4%
0.9	83.3%	78.2%	84.6%	79.3%	79.2%	80.0%

Supplementary Table S8: **The median sensitivity and precision values for the training-size experiment on the Arabidopsis datasets.** For each dataset, an increase in training-size resulting in an overall increase in sensitivity, supporting the conclusion that deeper sequencing experiments can yield more information on regulatory interactions. The characteristics of the sampling data, such as sequencing depth and complexity also influence the sensitivity and precision values (as observed for replicate B).

Retain rate	D1A Sensitivity	D1A Precision	D1B Sensitivity	D1B Precision	D1C Sensitivity	D1C Precision
0.5	23.3%	100.0%	22.9%	100.0%	22.3%	96.4%
0.55	24.8%	100.0%	26.7%	100.0%	23.1%	96.6%
0.6	26.4%	100.0%	27.5%	100.0%	25.6%	93.9%
0.65	27.1%	100.0%	32.8%	100.0%	30.6%	90.2%
0.7	73.6%	95.0%	72.5%	95.0%	76.9%	92.1%
0.75	75.2%	95.1%	74.8%	95.2%	78.5%	92.2%
0.8	83.7%	93.9%	84.7%	93.3%	86.0%	88.9%
0.85	86.8%	93.3%	88.6%	92.1%	88.4%	88.4%
0.9	87.6%	90.4%	89.3%	89.3%	88.4%	85.6%
0.95	96.1%	85.5%	96.2%	83.4%	94.2%	81.4%
1	99.2%	82.1%	99.2%	78.3%	99.2%	79.0%

Supplementary Table S9: **Analysis of the retain rate parameter using the *A. Thaliana* D1 dataset.** The dual optimization problem for maximizing the increase in Se and minimizing the loss PPV is solved using the Se/PPV ratio, which for this dataset achieves its value at 0.85.

Species	*A. thaliana*	*A. trichopoda*	*O. sativa*	*T. aestivum*
A. thaliana	1	0.331	**2.54×10^{-4}**	**6.89×10^{-6}**
A. trichopoda		1	**1.93×10^{-7}**	**1.38×10^{-8}**
O. sativa			1	0.166
T. aestivum				1

Supplementary Table S12: **Results of the Kolmogorov-Smirnov test when evaluating the differences between MFE ratio distributions** of HC miRNA-mRNA interactions found in flower tissue in model and non-model organisms. The results highlight the significant differences observed between dicots (*A. thaliana* and *A. trichopoda*) and monocots (*O. sativa* and *T. aestivum*).

Position	χ2	MM	G:U	Gap
1	0.41	0.12	0.44	1
2	0.49	0.44	0.74	0.44
3	0.43	0.21	1	1
4	0.67	0.44	0.99	1
5	0.97	1	1	1
6	0.56	1	0.62	0.62
7	0.58	0.43	0.76	0.62
8	0.34	0.16	0.49	1
9	0.61	0.68	0.49	1
10	0.95	1	1	1
11	0.78	0.68	1	1
12	0.52	0.36	1	1
13	0.95	1	1	1
14	0.97	0.83	0.76	1
15	0.98	0.78	0.82	0.99
16	0.91	1	1	1
17	0.99	0.78	1	1
18	0.71	0.79	0.44	1
19	0.84	0.61	0.99	0.62
20	0.66	1	0.48	0.44
21	0.55	0.19	0.59	0.61

Supplementary Table S13: **χ^2 and Fisher's exact test significance results on the position-specific properties for conserved and species-specific miRNA-mRNA interactions in *A. trichopoda* leaf***.* The contribution of specific properties such as MM, Gaps and G:U is assessed using Fisher exact tests.

Position	χ2	MM	G:U	Gap
1	0.03	0.33	0	1
2	0.03	1	0	0.36
3	0.78	1	1	1
4	0.93	0.71	0.68	1
5	0.08	1	0.01	1
6	0.2	0.62	1	0.11
7	0.72	0.44	0.99	0.99
8	0.44	0.44	0.21	0.99
9	0.09	0.03	0.74	1
10	0.95	1	1	1
11	0.27	0.21	0.62	1
12	0.75	0.62	1	1
13	0.79	0.62	1	1
14	0.33	1	0.11	1
15	0.95	1	0.59	1
16	0.57	0.3	0.74	1
17	0.48	0.25	0.74	1
18	0.78	0.99	0.49	1
19	0.9	0.61	1	1
20	0.47	0.15	1	1
21	0.38	0.88	0.19	0.36

Supplementary Table S14: **χ^2 and Fisher's exact test significance results on the position-specific properties for conserved and species-specific miRNA-mRNA interactions in *A. trichopoda* flower***.* The contribution of specific properties such as MM, Gaps and G:U is assessed using Fisher exact tests.

Position	χ2	MM	G:U	Gap
1	0.24	0.24	0.09	1
2	0.37	0.72	0.36	0.35
3	0.95	1	1	1
4	0.63	0.44	1	1
5	0.87	0.68	1	1
6	0.7	0.68	1	1
7	0.98	1	1	1
8	0.26	0.62	0.16	1
9	0.36	0.68	0.21	1
10	0.19	0.36	0.21	0.99
11	0.87	0.72	1	1
12	0.85	1	1	1
13	0.52	0.72	0.44	0.99
14	0.93	1	0.74	1
15	0.55	1	0.25	1
16	0.72	0.68	1	1
17	0.87	0.56	0.99	0.99
18	0.93	1	1	0.99
19	0.24	0.21	0.33	1
20	0.89	1	0.81	1
21	0.68	0.55	0.4	1

Supplementary Table S15: **χ^2 and Fisher's exact test significance results on the position-specific properties for conserved and species-specific miRNA-mRNA interactions in *G. max* leaf***.* The contribution of specific properties such as MM, Gaps and G:U is assessed using Fisher exact tests.

Position	χ2	MM	G:U	Gap
1	0.13	0.1	0.44	1
2	0.04	0.11	1	0.03
3	0.79	1	0.62	1
4	1	1	1	1
5	0.78	0.49	1	1
6	0	0.11	0	1
7	0.27	0.09	1	1
8	0.3	0.36	0.36	1
9	0.07	0	0.85	0.99
10	0.79	1	0.62	1
11	0.79	1	0.62	1
12	0.02	0.03	0.13	1
13	0.93	1	1	1
14	0	0	0.36	1
15	**0.05**	0.06	0.21	1
16	0.79	1	0.62	1
17	0.73	0.33	1	0.72
18	0.67	0.99	0.74	0.62
19	0.7	0.37	1	1
20	0.03	0.01	0.83	0.63
21	0	0	0	0.44

Supplementary Table S16: **χ^2 and Fisher's exact test significance results on the position-specific properties for conserved and species-specific miRNA-mRNA interactions in *O. sativa* inflorescence**. The contribution of specific properties such as MM, Gaps and G:U is assessed using Fisher exact tests.

Position	χ2	MM	G:U	Gap
1	0	0.34	0	1
2	0.01	0.72	0.16	0
3	0.01	0.16	0	0.99
4	0.31	0.21	1	1
5	0.4	0.36	0.62	1
6	0.12	0.06	0.21	0.99
7	0.6	0.72	0.37	1
8	0.07	0.62	0.03	1
9	0.61	0.49	0.78	0.36
10	0.57	0.21	1	0.99
11	0.79	1	0.36	0.99
12	0	0	0.27	1
13	0.16	0.21	0.36	1
14	0	0	0.44	1
15	0	0	0.21	1
16	0.02	0	0.74	0.99
17	**0.05**	0.09	0.02	0.62
18	0	0	0.03	1
19	0.42	0.44	0.62	1
20	0.07	0.03	0.53	1
21	0.18	0.06	0.21	1

Supplementary Table S17: **χ^2 and Fisher's exact test significance results on the position-specific properties for conserved and species-specific miRNA-mRNA interactions in *T. aestivum* spikes***.* The contribution of specific properties such as MM, Gaps and G:U is assessed using Fisher exact tests.

Dataset	*Allen et al.* miRNAs	*Allen et al.* interactions	Inferred miRNAs	Inferred interactions	Difference in miRNAs	Difference in interactions
D4A	70	203	87	272	17	69
D4B	66	174	79	208	13	34
D5	143	2118	190	2842	47	724
D6	42	149	46	161	4	12
D7	91	1257	193	2040	102	783

Supplementary Table S18: **Frequency summary on the count of miRNAs and corresponding interactions determined using either the *Allen et al.* rules or the inferred rules for non-model organisms.** The difference in number of miRNAs and their interactions when comparing the *Allen et al.* criteria and the criteria inferred by PAREameters using a retain rate of 1. All of the *Allen et al.* reported interactions are a subset of the inferred criteria reported interactions when using a retain rate of 1.

Dataset	D1B				D1C			
Position	χ^2	MM	GU	Gap	χ^2	MM	GU	Gap
1	0.97	0.69	0.87	0.99	0.99	1	1	0.99
2	0.72	0.62	1	0.78	0.86	0.99	1	0.53
3	0.95	1	1	1	1	1	1	1
4	0.91	0.68	0.99	1	0.99	1	0.99	1
5	1	1	1	1	1	1	1	1
6	0.93	1	1	1	1	1	1	1
7	0.93	1	1	1	0.95	0.99	0.81	1
8	0.91	1	0.49	0.99	0.91	1	0.49	0.99
9	0.96	0.72	0.74	0.99	0.97	1	0.99	0.99
10	0.88	1	0.62	0.99	0.88	1	0.62	0.99
11	0.94	1	1	1	0.95	0.78	1	1
12	0.94	1	0.68	0.99	0.93	1	1	1
13	0.95	1	1	1	0.95	1	0.62	0.99
14	0.94	1	1	1	0.25	0.11	0.36	0.99
15	0.99	1	1	1	0.93	1	0.56	1
16	0.92	1	1	0.99	0.4	1	0.21	0.99
17	0.99	0.83	1	0.99	0.99	1	0.84	0.99
18	0.91	0.76	0.99	1	0.99	1	1	1
19	0.99	0.81	1	1	0.97	0.81	1	1
20	0.99	1	1	1	0.99	1	0.99	1
21	0.9	0.88	0.8	1	0.95	1	0.99	1

Supplementary Table S19: **χ^2 and Fisher's exact test significance results on the position-specific properties for HC interactions in D1B and D1C versus *A. thaliana* D1A (WTA) dataset**. The contribution of specific properties such as MM, Gaps and G:U is assessed using Fisher exact tests.

Dataset	D1B				D1C			
Position	χ^2	MM	GU	Gap	χ^2	MM	GU	Gap
1	0.92	0.64	0.82	1	0.55	0.34	0.32	1
2	0.91	1	1	0.8	0.9	1	0.74	0.8
3	0.82	1	0.8	1	0.24	1	0.1	1
4	0.71	1	0.62	1	0.6	1	0.36	1
5	0.95	0.99	1	1	0.98	1	1	1
6	0.98	1	1	1	0.79	0.68	0.99	1
7	0.94	1	1	1	0.79	0.63	0.49	0.99
8	0.72	1	0.44	1	0.63	1	0.44	1
9	0.7	1	0.37	1	1	1	1	1
10	0.75	0.62	1	1	0.92	0.99	1	1
11	0.93	0.74	1	1	0.57	0.25	1	1
12	0.43	0.16	1	1	0.6	0.4	0.74	0.62
13	0.9	1	0.82	1	0.03	1	0	0.62
14	0.74	0.37	0.72	0.99	0.24	0.06	1	1
15	0.75	0.66	0.74	1	0.98	1	1	1
16	0.87	1	0.78	1	0.75	0.72	0.78	1
17	0.98	0.99	0.99	0.99	0.85	0.99	0.56	1
18	0.93	0.64	1	0.72	0.8	0.81	1	0.68
19	0.86	0.54	0.99	1	0.81	0.64	1	0.68
20	0.95	1	0.67	1	0.43	0.18	1	0.99
21	0.97	1	0.8	1	0.93	0.74	0.8	1

Supplementary Table S20: **χ^2 and Fisher's exact test significance results on the position-specific properties for LC interactions in D1B and D1C versus *A. thaliana* D1A (WTA) dataset**. The contribution of specific properties such as MM, Gaps and G:U is assessed using Fisher exact tests.
